# Association Between Systemic Lupus Erythematosus and Cancer Morbidity and Mortality: Findings From Cohort Studies

**DOI:** 10.3389/fonc.2022.860794

**Published:** 2022-05-04

**Authors:** Min Zhang, Yizhou Wang, Yutong Wang, Ye Bai, Dongqing Gu

**Affiliations:** ^1^ School of Public Health and Management, Chongqing Medical University, Chongqing, China; ^2^ Department of Pathology, The Third Hospital of Mianyang, Sichuan Mental Health Center, Mianyang, China; ^3^ Department of Epidemiology and Medicine, West China School of Public Health and West China Fourth Hospital, Sichuan University, Chengdu, China; ^4^ Department of Infectious Diseases, First Affiliated Hospital, Army Medical University, Chongqing, China

**Keywords:** systemic lupus erythematosus, cancer, meta-analysis, cohort study, Mendelian randomization

## Abstract

**Background:**

Observational studies suggested that systemic lupus erythematosus (SLE) might be associated with increased cancer incidence and cancer-related death, however, the results are inconsistent. We aim to comprehensively estimate the causal relationships between SLE and cancer morbidity and mortality using a meta-analysis of cohort studies and Mendelian randomization.

**Methods:**

A systematic search was conducted using PubMed to identify cohort studies published before January 21, 2021. Meta-analysis was performed to calculate relative risk (RR) and corresponding 95% confidence intervals (CI). In addition, we further evaluated the potentially causal relationships identified by cohort studies using two-sample Mendelian randomization.

**Results:**

A total of 48 cohort studies involving 247,575 patients were included. We performed 31 main meta-analysis to assess the cancer risk and three meta-analyses to evaluate cancer mortality in SLE patients. Through meta-analyses, we observed an increased risk of overall cancer (RR=1.62, 95%CI, 1.47-1.79, *P*<0.001) and cancer-related death (RR=1.52, 95%CI, 1.36-1.70, *P*<0.001) in patients with SLE. Subgroup analysis by site-specific cancer showed that SLE was a risk factor for 17 site-specific cancers, including six digestive cancers (esophagus, colon, anus, hepatobiliary, liver, pancreatic), five hematologic cancers (lymphoma, Hodgkin’s lymphoma, non-Hodgkin lymphoma, leukemia, multiple myeloma), as well as cancer in lung, larynx, cervical, vagina/vulva, renal, bladder, skin, and thyroid. In addition, further mendelian randomization analysis verified a weakly association between genetically predisposed SLE and lymphoma risk (odds ratio=1.0004, *P*=0.0035).

**Conclusions:**

Findings from our study suggest an important role of SLE in carcinogenesis, especially for lymphoma.

**Systematic Review Registration:**

https://www.crd.york.ac.uk/PROSPERO/, CRD42021243635.

## 1 Introduction

Systemic lupus erythematosus (SLE) is an autoimmune disease characterized by the presence of nuclear autoantibodies which could cause immune complexes formation, and thus resulting in inflammation of multiple organs (1). Globally, the prevalence of SLE has been estimated to be 30-150 per 100,000, and incidence ranges from 2.2 to 23.1 per 100,000 each year (1). It is reported that there are approximately 500,000 SLE patients in Europe and 250,000 in the USA (2, 3). The clinical presentation of SLE is heterogeneous and can involve one or more organs, including the skin, joints, kidneys, blood cells, and nervous system, taking a chronic or relapsing and remitting disease course (4).

In past decades, various observational studies have investigated the relationship between SLE and cancers. Patients with SLE generally carry an increased risk of developing cancers. However, the relationship between SLE and cancer is complex. Recent epidemiologic evidence have suggested that there is an increased risk of some malignancies, including lung cancer, liver cancer, cervical cancer, and especially some hematologic cancers, such as non-Hodgkin lymphoma, Hodgkin lymphoma and leukemia among patients with SLE (5–10). The highest relative risk (RR) for lymphoma is associated with primary Sjogren’s syndrome (SS), followed by SLE and rheumatoid arthritis (RA), indicating a disease-specific risk profile (11, 12). However, the role of immunosuppressive medications in the development of cancer and/or lymphoma in SLE patients is still controversial (13, 14). On the contrary, some studies have found a decreased risk of some hormone-sensitive cancers, such as breast, ovarian, and endometrial cancer, in SLE patients (15–17). Importantly, the causal role of SLE in cancer is weak since inference from observational studies (especially cross-sectional and case-control studies) is limited by residual or unmeasured confounding and other biases such as reverse causation and detection bias (18, 19).

Meta-analysis of cohort studies is a useful approach to summarize data from multiple studies. This method can increase statistical power, and is a major criterion for determining causality (20, 21). In recent years, Mendelian randomization analysis was another method that has been widely used to assess potential causal estimates of various risk factors with outcomes of interest. This approach has the advantage over conventional observational studies of minimizing potential biases by using genetic markers as instrumental variables of environmental risk factors (22). Some meta-analyses (23, 24) have already assessed the cancer risk in SLE patients, but they do not estimate cancer mortality. Using Mendelian randomization analysis, Peng et al. have shown SLE might as a causal risk factor for lung cancer (25), however, the causal association between SLE and other tumors remains to be further evaluated.

In the present study, we systematically investigate the correlation between SLE and overall cancer and site-specific cancers risk by conducting a meta-analysis of published cohort studies. Furthermore, we estimate the cause-specific standard mortality ratio (SMR) for cancer in patients with SLE. In addition, utilizing SLE-related SNPs as instrumental variables identified by the GWAS with the largest sample size, we investigated the correlation between genetically predisposed SLE and site-specific cancer risk using data from MR-Base.

## 2 Materials and Methods

Since summary statistics of published studies were used, no additional ethical approval from an institutional review board was required.

### 2.1 Meta-Analysis of Cohort Studies

Our methodology for the meta-analysis followed the guidelines proposed by the Preferred Reporting Items for Systematic Reviews and Meta-Analyses (PRISMA) statement and Meta-analysis Of Observational Studies in Epidemiology (MOOSE) (26, 27). The review protocol of this meta-analysis was registered in the International prospective register of systematic reviews (PROSPERO, registration ID: CRD42021243635), https://www.crd.york.ac.uk/PROSPERO/.

#### 2.1.1 Literature Search

**Figure 1:**
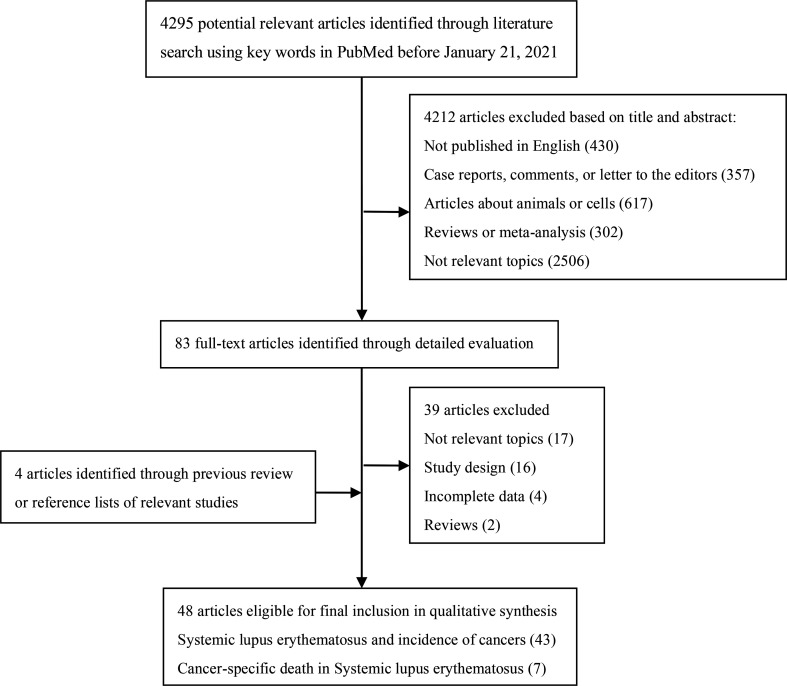
Flow diagram of literature search and study selection.

We searched PubMed to identify cohort studies that investigated the association between SLE and cancer risk published before January 21, 2021 (**Figure 1**). The keywords were “systemic lupus erythematosus OR lupus OR SLE” combined with “cancer OR adenocarcinoma OR carcinoma OR tumor OR malignancy OR neoplasm” as query terms. The title and abstract of studies, or full text if necessary was reviewed to identify all relevant publications. In addition, reference lists of all included studies as well as reviews and meta-analyses were manually screened for extra potential studies.

#### 2.1.2 Eligibility Criteria

We only included studies in our analyses as follows: (1) cohort studies that quantitatively investigated this association; (2) exposure to SLE; (3) the outcome of interest was cancer; (4) providing risk estimates (such as relative risks (RRs), standardized incidence ratios (SIRs), odds ratios (ORs) or hazard ratios (HRs) with 95% confidence intervals (CIs) or data to calculate them; (5) written in English language. For multiple publications using the same study population, the latest publication or publication with the largest sample size was included. Abstracts, reviews, letters, case reports and studies that did not provide sufficient data to calculate the risk estimates were excluded.

#### 2.1.3 Data Extraction

Two authors (MZ and YZW) independently performed literature screening and data extraction, and the disagreement was resolved by the corresponding author (DG). Extract the characteristic data of the study and the effect measure data of the interested results.

#### 2.1.4 Risk of Bias and Quality Assessment of the Included Literature

The Newcastle-Ottawa scale (NOS) (28) was used to assessed the methodological quality of each cohort study by two authors independently (MZ and YZW). The Newcastle-Ottawa scale evaluates study quality based on a ‘star scale system’ on three criteria: (i) selection of the study groups; (ii) the comparability of the groups; (iii) ascertainment of outcomes of interest.

#### 2.1.5 Statistical Analysis

Statistical analyses were done using Stata version 15 (Stata, College Station, TX, USA). We combined relative risks (RRs) of overall cancer and sites-specific cancer in SLE patients using a random-effects meta-analysis. For studies not containing RR, standard incidence ratio (SIR), odds ratio (OR) or hazard ratio (HR) were treated as the substitute of RR in the current study. We also performed subgroup meta-analyses based on region (Europe, America, or Asia). Furthermore, we estimate the cause-specific standard mortality ratio (SMR) for cancer in patients with SLE. We used the Cochran’s Q statistic to test for heterogeneity and the *I^2^* statistic to quantify heterogeneity across studies. Potential publication bias was assessed using Begg’s and Egger’s approaches. Sensitivity analyses were performed to evaluate the robustness of the results. We considered *P* values of less than 0.10 in tests of heterogeneity and publication bias, and *P* values of less than 0.05 in the meta-analyses to be statistically significant. All tests were two-sided, with the exception of tests of heterogeneity and publication bias.

### 2.2 Mendelian Randomization Analysis

The positive associations identified by the meta-analysis were further confirmed by two-sample Mendelian randomization analysis. The SNPs identified by the largest GWAS in populations of European ancestry were selected as instrumental variables respectively. We calculated the proportion of variance (R^2^) explained in the risk factor by the SNP(s) and the strength of the instrument (*F*-statistic). Details of methodology for SNP selection, and calculation of the R^2^ and *F*-statistic are presented in the **Supplementary Methods**.

We used the inverse variance weighted (IVW) fixed-effect method as the main method to estimate the effect of genetically predicted SLE on sites-specific cancer. Other Mendelian randomization methods including MR-Egger, weighted median, and weighted mode method were used to check the consistency of the direction of effect estimates. All analyses were conducted using the TwoSampleMR and MRInstruments R packages, curated by MR-Base platform (www.mrbase.org). All tests were two-sided, and *P* values of less than 0.05 were considered statistically significant unless stated otherwise.

## 3 Results

### 3.1 Literature Search

The literature search and selection process were presented in **Figure 1**. The comprehensive search generated 4295 potentially relevant studies, of which 4212 articles were excluded based on title and abstract review. We then examined full texts of the remaining 83 studies and excluded 39 of them. To be specific, 17 studies with irrelevant topics, 16 studies in which the study design was not cohort, four studies with incomplete data to calculate the estimates, and two studies being reviews were excluded. Additionally, four articles were included through previous review or reference lists of relevant studies. Finally, 48 eligible articles were included in the meta-analysis.

### 3.2 Characteristics of the Included Studies

The characteristics of the included studies were summarized in **Supplementary Table S1**. 48 articles with a total of 247,575 patients with SLE (more than 178,332 females and 19,900 males) were represented. Among them, 43 studies estimated the cancer incidence in SLE patients with the follow-up period ranging from 1,000 person-years to 157,969 person-years or from 4.7 years to 35.3 years. Seven studies estimated cancer-specific death in patients with SLE, with the follow-up period ranging from 48 person-years to 91,669 person-years or from 8.1 years to 11.9 years. In addition, a total of 30 human cancers were systematically divided into six systemic groups (digestive cancers, respiratory cancers, reproductive cancers, urinary cancers, hematopoietic cancers, and other cancers). As to the definition of SLE, 19 articles based on the American College of Rheumatology criteria (ACR), six articles were based on the American Rheumatism Association criteria (ARA).

According to the NOS (**Supplementary Table S2**), the mean number of stars for the 48 publications was 7.85, and 43 publications (89.5%) got more than eight stars, suggesting a high quality of the included studies.

### 3.3 Risk of Cancer in SLE

**Figure 2:**
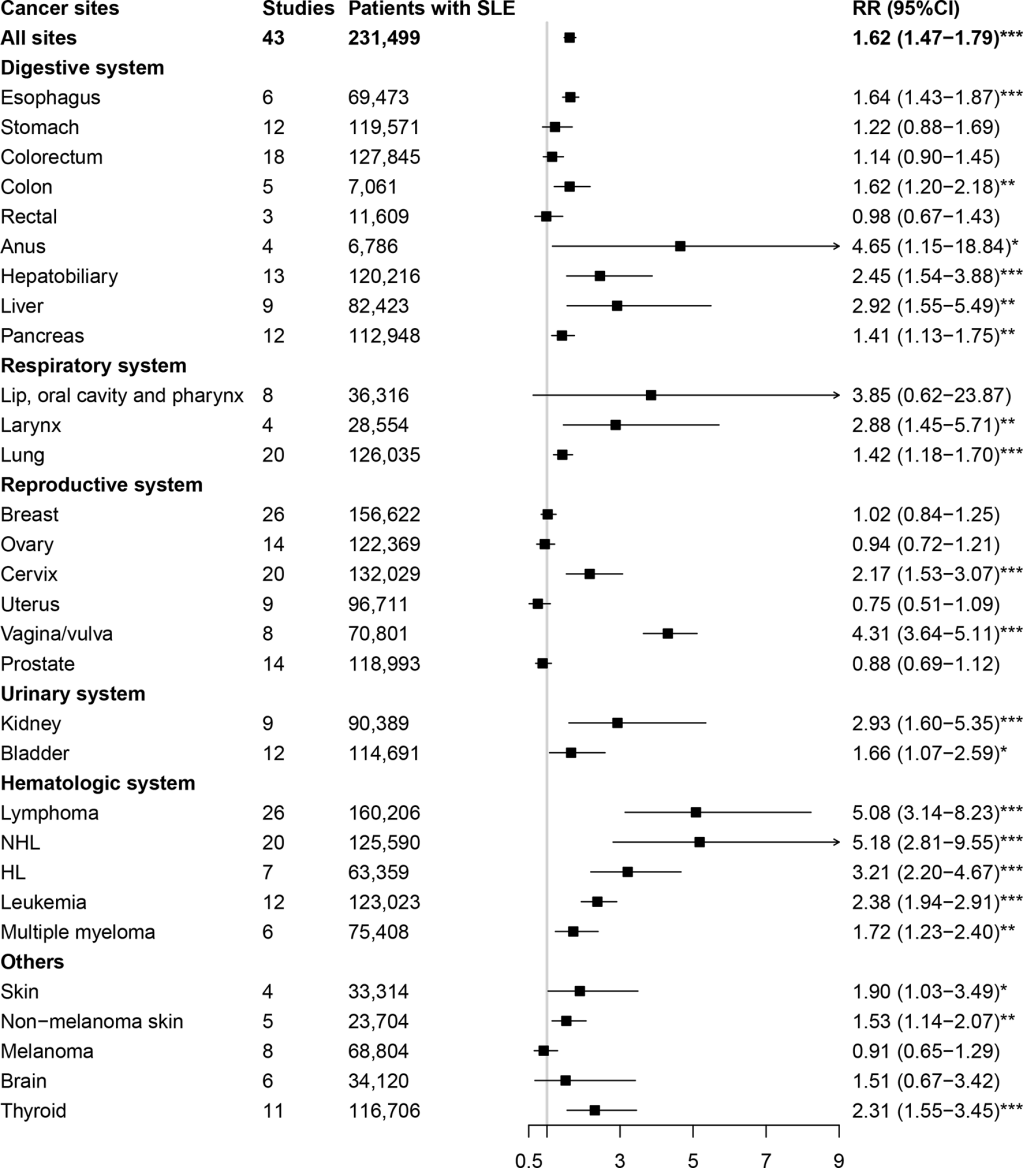
Overall cancer and site-specific cancer risk in patients with systemic lupus erythematosus. SLE, systemic lupus erythematosus; NHL, non-Hodgkin lymphoma; HL, Hodgkin lymphoma. ^*^ *P*-value < 0.05; ^**^ *P-*value < 0.01; ^***^ *P*-value < 0.001.

The relationships between SLE and cancers were shown in **Table 1** and **Figure 2**. Our results suggested an increased cancer risk in patients with SLE (RR=1.62, 95% CI, 1.47-1.79). Site-specific analysis suggested that SLE were associated with an increased risk of lymphoma, Hodgkin’s lymphoma, non-Hodgkin Lymphoma, leukemia, multiple myeloma, as well as esophagus, colon, anal, hepatobiliary, liver, pancreatic, larynx, lung, cervical, vagina/vulva, renal, bladder, skin (non-melanoma), and thyroid cancers. However, no significant associations were observed between SLE and cancers of stomach, colorectum, rectal, lip, oral cavity and pharynx, breast, ovary, uterus, prostate, melanoma, and brain.

**Table 1:** Meta-analysis of the association between systemic lupus erythematosus and cancer risk.

Cancer sites	Studies	Patients with SLE	Cancer morbidity		Heterogeneity	
			RR (95%CI)	*P* value	*I* ^2^	*P* value
**All sites**	**43**	**231,499**	**1.62 (1.47-1.79)**	**1.18×10^-21^**	**94.4%**	**<0.001**
**Digestive system**						
Esophagus	6	69,473	1.64 (1.43-1.87)	8.68×10^-13^	0.0%	0.84
Stomach	12	119,571	1.22 (0.88-1.69)	0.225	79.3%	<0.001
Colorectum	18	127,845	1.14 (0.90-1.45)	0.284	86.3%	<0.001
Colon	5	7,061	1.62 (1.20-2.18)	0.002	0.0%	0.983
Rectal	3	11,609	0.98 (0.67-1.43)	0.919	0.0%	0.814
Anus	4	6,786	4.65 (1.15-18.84)	0.031	90.5%	<0.001
Hepatobiliary	13	120,216	2.45 (1.54-3.88)	1.39×10^-04^	96.2%	<0.001
Liver	9	82,423	2.92 (1.55-5.49)	0.001	92.7%	<0.001
Pancreas	12	112,948	1.41 (1.13-1.75)	0.002	75.0%	<0.001
**Respiratory system**						
Lip, oral cavity and pharynx	8	36,316	3.85 (0.62-23.87)	0.147	97.5%	<0.001
Larynx	4	28,554	2.88 (1.45-5.71)	0.002	35.8%	0.198
Lung	20	126,035	1.42 (1.18-1.70)	1.39×10^-04^	72.8%	<0.001
**Reproductive system**						
Breast	26	155,622	1.02 (0.84-1.25)	0.821	93.6%	<0.001
Ovary	14	122,369	0.94 (0.72-1.21)	0.619	57.7%	0.004
Cervix	20	132,029	2.17 (1.53-3.07)	1.30×10^-05^	94.7%	<0.001
Uterus	9	96,711	0.75 (0.51-1.09)	0.134	80.7%	<0.001
Vagina/vulva	8	70,801	4.31 (3.64-5.11)	7.48×10^-64^	10.9%	0.345
Prostate	14	118,993	0.88 (0.69-1.12)	0.289	38.5%	0.07
**Urinary system**						
Kidney	9	90,389	2.93 (1.60-5.35)	4.83×10^-04^	94.2%	<0.001
Bladder	12	11,4691	1.66 (1.07-2.59)	0.024	85.9%	<0.001
**Hematologic system**						
Lymphoma	26	160,206	5.08 (3.14-8.23)	3.59×10^-11^	99.4%	<0.001
NHL	20	125,590	5.18 (2.81-9.55)	1.29×10^-07^	99.6%	<0.001
HL	7	63,359	3.21 (2.20-4.67)	1.20×10^-09^	0.0%	0.499
Leukemia	12	123,023	2.38 (1.94-2.91)	4.86×10^-17^	46.4%	0.039
Multiple myeloma	6	75,408	1.72 (1.23-2.40)	0.001	0.4%	0.413
**Others**						
Skin	4	33,314	1.90 (1.03-3.49)	0.039	49.3%	0.116
Non-melanoma skin	5	23,704	1.53 (1.14-2.07)	0.005	0.0%	0.586
Melanoma	8	68,804	0.91 (0.65-1.29)	0.607	57.9%	0.02
Brain	6	34,120	1.51 (0.67-3.42)	0.324	73.0%	0.002
Thyroid	11	116,706	2.31 (1.55-3.45)	4.31×10^-05^	94.3%	<0.001

SLE, systemic lupus erythematosus; RR, relative risk ratio; CI, confidence interval; NHL, non-Hodgkin lymphoma; HL, Hodgkin lymphoma.

The results of subgroup analysis by geographic region were presented in **Table 2**. Patients with SLE were associated with an increased risk of overall cancers in Europe (RR=1.66, 95% CI, 1.35-2.03), America (RR=1.58, 95% CI, 1.19-2.09), and Asia (RR=1.57, 95% CI, 1.39-1.77). In region of Europe, we observed an increased risk of lymphoma, Hodgkin’s lymphoma, non-Hodgkin Lymphoma, leukemia, as well as colon, anal, hepatobiliary, liver, pancreatic, larynx, lung, cervical, vagina/vulva, bladder, and skin (non-melanoma) cancers in patients with SLE. In region of America, we observed an increased risk of liver cancer, lymphoma, Hodgkin’s lymphoma and non-Hodgkin Lymphoma, whereas a deceased risk of prostate cancer in patients with SLE. In Asia, patients with SLE were associated with an increased risk of esophagus, hepatobiliary, liver, lip, oral cavity and pharynx, lung, cervical, skin (non-melanoma), brain and thyroid cancers, as well as lymphoma, non-Hodgkin lymphoma, and leukemia.

**Table 2:** Results of Subgroup analysis by geographic region in the meta-analysis.

Cancer sites	European countries			Asian countries			American countries		
	Studies	Patients^*a*^	RR (95%CI)	Studies	Patients^*a*^	RR (95%CI)	Studies	Patients^*a*^	RR (95%CI)
**All sites**	21	59,484	1.66 (1.35-2.03)^***^	12	88,630	1.57 (1.39-1.77)^***^	5	54,370	1.58 (1.19-2.09)^**^
**Digestive system**									
Esophagus	3	11,609	1.86 (0.84-4.11)	2	27,386	1.62 (1.41-1.86)^***^			
Stomach	5	13,683	1.12 (0.77-1.62)	4	49,454	1.32 (0.73-2.38)			
Liver	4	8,081	4.49 (1.43-14.08)^*^	2	23,166	1.39 (1.00-1.91)^*^	2	34,767	4.37 (1.82-10.46)^**^
Hepatobiliary	8	18,488	3.51 (1.87- 6.59)^***^	4	50,552	1.60 (1.31-1.97)^***^			
Pancreas	7	17,659	1.73 (1.31-2.28)^***^	3	48,402	1.10 (0.82-1.48)			
Colorectum	9	25,065	1.03 (0.83-1.27)	5	51,604	0.99 (0.77-1.28)	2	34,767	2.01 (0.42-9.64)
Colon	5	7,061	1.62 (1.20-2.18)^**^						
Rectal	3	11,609	0.98 (0.67-1.43)						
Anus	4	6,786	4.65 (1.15-18.84)^*^						
**Respiratory system**									
Lip, oral cavity and pharynx	5	7,653	1.87 (0.88-3.96)	2	24,374	1.71 (1.04-2.79)^*^			
Lung	10	18,511	1.65 (1.26-2.16)^***^	4	39,014	1.37 (1.10-1.71)^**^	3	35,383	1.40 (0.68-2.91)
Larynx	3	7,538	3.07 (1.28-7.35)^*^						
**Reproductive system**									
Prostate	6	7,718	1.53 (0.66-3.53)	4	50,552	1.11 (0.58-2.12)	2	34,767	0.68 (0.50-0.93)^*^
Breast	12	16,650	0.82 (0.64-1.06)	5	51,604	1.12 (0.83-1.51)	5	31,658	1.37 (0.95-1.98)
Ovary	6	13,244	0.85 (0.57-1.26)	4	48,402	1.07 (0.61-1.88)	2	34,767	1.34 (0.47-3.83)
Cervix	12	20,459	2.39 (1.57-3.62)^***^	4	48,627	2.43 (1.41-4.18)^**^	2	34,767	2.90 (0.10-81.56)
Uterus	4	12,891	0.97 (0.72-1.29)	2	27,386	0.71 (0.18-2.79)			
Vagina/vulva	4	2,604	7.06 (3.03-16.43)^***^						
**Urinary system**									
Kidney	4	7,220	2.71 (0.81-9.10)	3	48,402	1.87 (0.69-5.10)	2	34,767	5.78 (0.84-40.08)
Bladder	5	8,941	1.81 (1.05-3.12)^*^	4	49,316	2.46 (0.65-9.29)			
**Hematologic system**									
Lymphoma	13	23,743	5.41 (2.77-10.55)^***^	6	66,785	6.19 (4.86-7.89)^***^	3	35,383	4.99 (1.74-14.30)^**^
NHL	10	21,603	4.81 (3.65-6.34)^***^	5	45,769	6.18 (4.58-8.33)^***^	2	31,094	2.73 (2.23-3.35)^***^
HL	3	6,309	4.71 (2.00-11.07)^***^				2	31,094	4.41 (1.06-18.34)^*^
Leukemia	5	8,838	4.81 (1.46-15.83)^*^	4	57,751	2.63 (2.16-3.19)^***^			
Multiple myeloma	3	7,505	1.45 (0.74-2.85)						
**Others**									
Non-melanoma skin	4	8,081	1.68 (1.22-2.32)^**^						
Skin	2	535	2.21 (0.29-16.99)	2	32,779	1.67 (1.55-1.80)^***^			
Brain	4	6,734	0.77 (0.39-1.55)	2	27,386	2.91 (1.77-4.79)^***^			
Thyroid	3	6,529	1.29 (0.31-5.41)	4	49,454	1.75 (1.20-2.55)^**^	2	34,767	7.17 (0.499-104.96)
Melanoma	4	8,081	0.93 (0.50-1.73)				2	34,767	0.98 (0.47-2.01)

a Patients with systemic lupus erythematosus; NHL, non-Hodgkin lymphoma; HL, Hodgkin lymphoma.

^*^P value < 0.05.

^**^P value < 0.01.

^***^P value < 0.001.

### 3.4 Cancer-Specific Mortality in SLE

**Figure 3:**
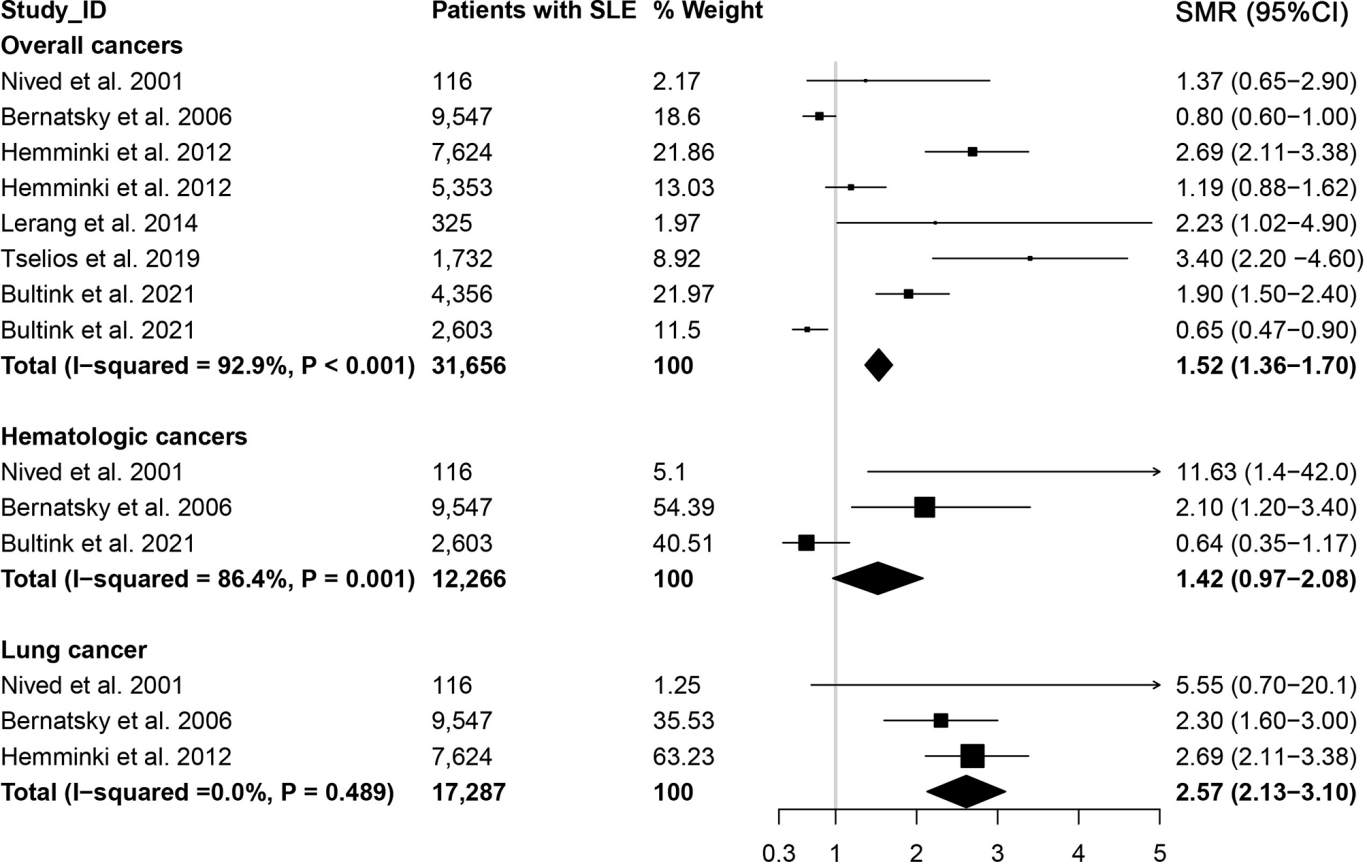
Overall cancer and site-specific cancer mortality in patients with systemic lupus erythematosus.

We also estimated the cancer-specific death in patients with SLE, and the results were presented in **Figure 3**. The cause-specific SMR was higher for overall cancer (SMR=1.52, 95% CI, 1.36-1.70) in patients with SLE, particularly for lung cancer (SMR=2.57, 95% CI, 2.13-3.10). However, the cause-specific SMR for hematologic cancer was 1.42 (95% CI, 0.97-2.08).

### 3.5 Heterogeneity and Sensitivity Analysis

As shown in **Table 1** and **Figure 3**, we totally conducted 34 meta-analyses to investigate the associations between SLE and cancer (or cancer-specific) morbidity and mortality. The heterogeneity of 64.7% relationships (22/34) was relatively high (*I* ^2^>50%), 11.8% relationships (4/34) showed moderate heterogeneity (25%> *I* ^2^ <50%), and 23.5% relationships (8/34) showed no/little heterogeneity (*I* ^2^ <25%). However, sensitivity analysis suggested that most associations did not significantly change when a single was excluded (data not shown).

### 3.6 Mendelian Randomization Results

The significant associations in European ancestry were further estimated by two-sample Mendelian randomization analysis. The instrumental variable of SLE was constructed using 69 SNPs (**Supplementary Table S3**), explaining approximately 34% of the heritability totally, and the *F*-statistic was 172.36 (*F*>100). We utilized the summary-level statistics for colon cancer, pancreatic cancer, lung cancer, cervical cancer, bladder cancer, lymphoma, and non-melanoma skin cancer from the MR-Base. The details of outcomes were shown in **Supplementary Table S4**.

Being consistent with the findings in the meta-analysis, the results of Mendelian randomization analysis indicated a causal association between genetically predisposed SLE and lymphoma (OR=1.0004, 95% CI, 1.0001-1.0007, *P*=0.0035), whereas a decreased risk of bladder cancer (OR=0.9996, 95% CI, 0.9994-0.9998, *P*=0.00004) in European ancestry as shown in **Supplementary Table S5**, **Supplementary Figures S1, S2**. Furthermore, the MR-Egger and weighted median methods yielded similar results (**Supplementary Table S5**). However, no association was observed between genetically predisposed SLE and risk of colon, pancreatic, lung, cervical and non-melanoma skin cancer in European ancestry.

## 4 Discussion

Our meta-analysis included 48 cohort studies involving approximately 247,575 patients with SLE to estimate the relationship between SLE and cancers. The present study demonstrated that patients with SLE had a 62% elevated risk of overall cancer morbidity and a 52% increased risk of cancer-related death. In addition, our study yielded a significantly risk of site-specific cancers among patients with SLE, including esophagus, colon, anal, hepatobiliary, liver, pancreatic, larynx, lung, cervical, vagina/vulva, renal, bladder, skin (non-melanoma), and thyroid cancers, as well as lymphoma, Hodgkin’s lymphoma, non-Hodgkin Lymphoma, leukemia, and multiple myeloma. This comprehensive study will provide epidemiological evidence supporting the associations between SLE and cancer. These evidences could be utilized to drive public policies and to help guide personalized medicine to better manage patients with SLE and reduce associated cancer morbidity and mortality.

Furthermore, our study used 69 SNPs identified by the largest GWAS in European ancestry as the final genetic variants, which can explain over 34% of the heritability totally, suggesting a strong instrumental variable for analyzing the causal relation between SLE and cancer risk. Using the Mendelian randomization approach, our study provided weak evidence for a possible causal association between SLE and lymphoma risk in European ancestry. As far as we know, our study provided the most comprehensive and latest evidence for assessing the causality between SLE and site-specific cancers risk through meta-analysis of cohort studies and Mendelian randomization analysis.

At present, the exact etiology underlying the attribution of SLE to cancer risk remains unclear. SLE is a chronic systemic autoimmune disease which could induce chronic multiorgan inflammatory lesion. The SLE-induced chronic inflammation may also promote the tissue injury, leading to the cancer development (29). Chemokines and/or cytokines [such as C-X-C motif chemokine ligand 10 (CXCL10), interleukin 1β] are involved in the etiopathogenesis of SLE (30, 31), and also play an important role in the formation of peri-tumor inflammation which is the determinant of the tumor microenvironment (32, 33). Furthermore, the chronic inflammation causing cellular apoptosis may also induce changes of cancer-associated genes (34). On the other hand, immunosuppressive agents may also contribute to the onset of malignancies. It has been reported that immunosuppressive therapy, such as glucocorticoid and cyclophosphamide (CTX), could also induce cytotoxic effects and suppress immune surveillance. Then the immune dysregulation of SLE may lead to the cellular damage and mutation, thus resulting in carcinogenesis of various cancers (35–38). It has been found a significant elevation in cancer risk in multiple sclerosis (MS) patients treated with immunosuppressive drugs, similarly to the case of the SLE (39–41). The risk of malignancy in MS patients who have received disease-modifying therapies has also been examined. However, results have showed that there is no difference in cancer risk among those patients compared with the general population (41, 42). Overall, more studies are warranted to further investigate the underlying mechanism.

According to our study, hematologic cancers, especially non-Hodgkin lymphoma, is a common malignancy in SLE patients. Meta-analysis of cohort studies suggested that SLE patients had a 5.08-fold increased risk of lymphoma, 5.18-fold increased risk of non-Hodgkin lymphoma, and 3.21-fold increased risk of Hodgkin’s lymphoma. Moreover, both meta-analysis and Mendelian randomization analysis results revealed a potential causal relationship between SLE and lymphoma risk in European ancestry. Several potential mechanisms may account for the association between SLE and lymphoma. First, B cell and T cell activation play crucial roles in the pathogenesis of SLE and non-Hodgkin lymphoma, and the dysfunction of immune cells (such as B cell) in patients with SLE may lead to abnormal B cell activation and proliferation, resulting in B cell malignancies (43). Second, chronic inflammation might heighten lymphoma risk in autoimmune diseases like SLE (44). Third, SLE and lymphoma have similar genetic susceptibility or risk factors (e.g. Epstein-Barr virus infection) (45–48). Fourth, the use of immunosuppressants (e.g. cyclophosphamide) might lead to lymphoma by direct mutagenesis or by disturbing immune surveillance (36, 49). Also, the animal models suggested an interaction between SLE and lymphoma (50, 51).

Interestingly, although meta-analysis results suggested an increased risk of bladder cancer, inconsistent results were observed in Mendelian randomization analysis. There are some plausible hypotheses that might explain the elevated risk of bladder cancers in patients with SLE. In particular, the use of immunosuppressive agents has an influence on the risk of bladder cancer in SLE patients. Several case series suggested that immunosuppressive agents increased the risk of bladder cancer in patients with SLE (52–54). Otherwise, Bernasky et al. reported that SLE patients with immunosuppressive therapy had a 25% increased risk of bladder cancer (55). However, these studies may have insufficient statistical power due to small sample sizes, and the development of SLE in relation to bladder cancer may be by chance. Therefore, we can hardly determine the relationship between SLE and bladder cancer risk according to the present evidence, and more related observational studies and biological studies are needed in the future.

The limitations of this study include: first, the heterogeneity was high in most of the meta-analyses. Besides, some of the included studies in our meta-analysis were the retrospective studies, and the results might be confounded by recall bias and other factors. However, sensitive analysis was performed which suggested the results were stable. Second, some outcome data were not found in MR-Base, therefore, we did not perform the Mendelian randomization analysis to identify these potentially causal relationships. Third, results from the meta-analysis of cohort studies and Mendelian randomization analysis were inconsistent concerning the relationship between SLE and bladder cancer risk, so future well-designed cohort studies and Mendelian randomization analysis are needed to determine this association.

## 5 Conclusions

In conclusion, our meta-analysis of cohort studies suggests that patients with SLE are susceptible to overall cancers, particularly among Europeans. In addition, the overall cancer mortality was higher in patients with SLE, especially for lung cancer. Besides, a higher risk of site-specific cancers of esophagus, colon, anal, hepatobiliary, liver, pancreatic, larynx, lung, cervical, vagina/vulva, renal, bladder, skin (non-melanoma), and thyroid, as well as lymphoma, Hodgkin’s lymphoma, non-Hodgkin lymphoma, leukemia and multiple myeloma are observed among patients with SLE. This study highlights the important role of SLE in carcinogenesis.

## Data Availability Statement

The original contributions presented in the study are included in the article/**Supplementary Material**. Further inquiries can be directed to the corresponding author.

## Author Contributions

All authors contributed significantly to this work. DG designed the research study and wrote the first draft of the manuscript; MZ and YZW performed literature screening, data extraction, and quality assessment; YTW and YB analyzed the data and conducted the Mendelian randomization analysis. All authors interpret the results, reviewed, edited and approved the manuscript.

## Funding

This study was supported by the National Natural Science Foundation of China (81903393), and Chongqing Natural Science Foundation Program (cstc2020jcyj-msxmX0021). The funders had no role in study design, data collection and analysis, decision to publish, or preparation of the manuscript.

## Conflict of Interest

The authors declare that the research was conducted in the absence of any commercial or financial relationships that could be construed as a potential conflict of interest.

## Publisher’s Note

All claims expressed in this article are solely those of the authors and do not necessarily represent those of their affiliated organizations, or those of the publisher, the editors and the reviewers. Any product that may be evaluated in this article, or claim that may be made by its manufacturer, is not guaranteed or endorsed by the publisher.
