# Supplementary material for: Association Between Systemic Lupus Erythematosus and Cancer Morbidity and Mortality: Findings From Cohort Studies
Source: Front Oncol. 2022 May 4;12:860794. doi: 10.3389/fonc.2022.860794 (PMC9115099; doi:10.3389/fonc.2022.860794)
Supplement: Supplementary file 1 [file DataSheet_1.pdf]

Supplementary Materials to:

Association between systemic lupus erythematosus and cancer: findings from cohort studies and Mendelian randomization analysis

Min Zhang[1#], Yizhou Wang[2#], Yutong Wang[3], Ye Bai[4], Dongqing Gu[5]*

[1]Institute for Brain Science and Disease, Chongqing Medical University, Chongqing 400016, China.

[2]Department of Pathology, Affiliated Hospital and Third Hospital of Mianyang, Chongqing Medical University, Mianyang 621000, China

[3]Department of Epidemiology and Medicine, West China School of Public Health and West China Fourth Hospital, Sichuan University, Chengdu 610041, Sichuan, China

[4]School of Public Health and Management, Chongqing 400016, China.

[5]Department of Epidemiology and Biostatistics, First Affiliated Hospital, Army Medical University, Chongqing 400038, China

[#]These authors contributed equally to this work.

***Corresponding Author**

Dongqing Gu, M.D., Ph.D.

Department of Epidemiology and Biostatistics

First Affiliated Hospital

Army Medical University

30 Gaotanyan Street, Shapingba District, Chongqing 400038, China

Phone: 86-23-6875-4311, Fax: 86-23-6875-4311
E-mail: dongqing.gu@vip.163.com

Supplementary Methods

Mendelian Randomization Analysis

We searched for SLE in GWAS catalog (www.ebi.ac.uk/gwas) to identify the associations between SNPs and SLE. The SNPs identified by the largest GWAS in populations of European ancestry or Asian ancestry were used to conduct instrumental variable respectively. Eventually, we used SNPs identified by Bentham et al. as instrumental variable in European ancestry [1]. Inclusion criteria of the SNPs as follows: (1) independent loci: defined as $r^2<0.1$, and for a locus in which multiple SNPs in linkage disequilibrium were identified in published GWAS, we selected the SNP with the most significant association; (2) GWAS-significant P-value threshold of $<5x10^{-08}$; (3) having the rs numbers (or position information) of significant SNPs; (4) providing beta-coefficient (β), standard error (SE), and p value (or sufficient data to calculate them). After selecting the set of SNPs, we extracted the following information for each SNP-risk factor association: rs numbers, effect allele, other allele, effect allele frequency, beta-coefficient (β), standard error (SE), and p value (or sufficient data to calculate them). We removed any SNP that was missing this information. Finally, 69 SNPs remained as the final genetic variants in European ancestry (**Supplementary Table S3**).

For the SNP(s) extracted for use in the Mendelian Randomization analysis, we calculated the proportion of variance explained (R^2) in the risk factor by the SNP(s) and the strength of the instrument (F-statistic) in our study using the formulae from Yarmolinsky et al. [2]. The formulas to calculate R^2 and F-statistic were:

$R^2 = (2\times \beta^2 \times \mathrm{MAF} \times (1-\mathrm{MAF}))/(2 \times \beta^2 \times \mathrm{MAF} \times (1-\mathrm{MAF}) + (\mathrm{SE}(\beta))^2 \times (2 \times \mathrm{N}) \times \mathrm{MAF} \times (1-\mathrm{MAF}))$, where β is the effect size (beta coefficient) for a given SNP, MAF is the minor allele frequency, SE(β) is the standard error of the effect size, and N is the sample size of the GWAS for the SNP-risk factor association.

$\mathrm{F} = \mathrm{R}^2 \times (\mathrm{N}-1-\mathrm{k})/((1-\mathrm{R}^2) \times \mathrm{k})$, where R^2 is the proportion of variance explained in the risk factor by the genetic instrument, N is the sample size of the GWAS, k is the number of SNPs included in the instrument.

Supplementary Table S1. Characteristics of the included studies in the meta-analysis

SLE and cancer incidence

Authors	Sources of SLE patients	Follow-up (person-years average years)	SLE diagnosis	Number of SLE patients (gender)	Cancer site	Number of cancers	RR (95% CI)
Pettersson et al. 1992[3]	Fourth Department of Medicine-Helsinki University Central Hospital	2,340 person-years	ARA criteria	205 (182 F/23M)	All sites	15	2.6 (1.5-4.4)
					NHL	4	44 (11.9-111)
					Soft tissue sarcomas	2	49 (6.0-177)
					Breast	4	2.7 (0.7-6.8)
Sweeney et al. 1995[4]				219	All sites		1.4 (0.5-3.0)
					NHL		10 (0.13-56)
Abu-Shakra et al. 1996[5]	The University of Toronto Lupus Clinic Database	7,233 patient-years 24 years	ACR criteria	724 (627 F/97 M)	All sites	24	1.08 (0.70-1.62)
					Hematologic	6	4.12 (1.52-9.01)
					NHL	3	5.38 (1.11-1 5.70)
					Leukemia	2	3.07 (0.37-11.1)
					Lung	4	1.54 (0.42-3.94)
					Breast	4	0.7 (0.19-1.8)
					Gynecologic	4	1.42 (0.39-3.64)
					Colon	3	2.04 (0.42-5.96)
					Pancreas	2	6.15 (0.75-22.20)
					Kidney	1	2.63 (0.07-14.66)
Mellemkjaer et al. 1997[6]	Nationwide Danish Hospital Discharge Register	10,807 person-years 6.8 years	ACR criteria	1,585 (1,308 F/277 M)	All sites	102	1.30 (1.06-1.58)
					Oropharynx	1	0.9 (0.0-4.7)
					Digestive organs	19	1.1 (0.7-1.8)
					Esophagus	1	2.0 (0.0-10.9)
					Stomach	2	1.0 (0.1-3.6)
					Colorectum	10	1.1 (0.5-1.9)
					Liver	5	8.0 (2.6-18.6)
					Pancreas	1	0.5 (0.0-2.5)
					Respiratory organs	17	1.9 (1.1-3.1)
					Larynx	2	1.9 (1.1-3.1)
					Lung	15	1.0 (0.5-1.7)
					Breast	14	4.0 (0.5-14.3)
					Cervix	2	0.7 (0.1-2.5)
					Uterus	4	1.2 (0.3-3.1)

					Vagina/vulva	3	5.7 (1.2-16.6)
					Prostate	1	0.6 (0.0-3.3)
					Kidney	1	0.5 (0.0-2.9)
					Bladder	5	1.6 (0.5-3.7)
					Melanoma	1	1.0 (0.5-1.8)
					Non-melanoma skin	10	1 .5 (0.3-4.3)
					Brain/nervous system	3	5.2 (2.2-10.3)
					NHL	8	1.4 (0.0-7.7)
					Multiple myeloma	1	2.0 (0.4-5.7)
					Leukemia	1	34.1 (0.9-190)
					Other specified and unspecified sites[f]	1	3.7 (1.0-9.5)
					Others[g]	4	3.8 (0.1-21)
Sultan et al. 2000[7]	University College London Lupus Clinic Database	1,695 patient-years, 4.8	ARA criteria	297 (249 F/48 M)	All sites	16	1.16 (0.56-2.13)
					HL	1	17.82 (0.45-99.23)
					Lung	1	1.31 (0.03-7.30)
					Breast	3	1.06 (0.21-5.9)
					Cervix	1	4.19 (0.11-23.33)
					Anal	1	0.69 (0.02-3.84)
					Prostate	1	1.34 (0.03-7.46)
Goldman et al. 1998[8]	NA	4051	NA	616	All sites	30	2.0 (1.4-2.9)
					Lung	NA	3.1 (1.3-7.9)
					Breast	NA	2.9 (1.4-6.4)
					NHL	NA	1.5 (0.02-8.6)
					HL	NA	17.82 (0.45-99.23)
Cibere et al. 2001[9]	University-based Rheumatic Disease Unit	12 years	ACR criteria	297 (249 F /48 M)	All sites	27	1.59 (1.05 2.32)
					Breast	4	1.15 (0.31-2.95)
					Colorectum	1	0.63 (0.008-3.53)
					Gynecologic	4	2.42 (0.65-6.20)
					Cervix	3	8.15 (1.63-23.81)
					Ovary	1	1.96 (0.02-10.95)
					Hematologic	5	4.90 (1.57-11.43)
					NHL	4	7.01 (1.88-17.96)
					HL	1	12.19 (0.15-67.85)
					Hepatobiliary	1	5.55 (0.07-30.91)

					Lung	3	2.09 (0.42-6.11)
					Oropharynx	1	10.00 (0.13-55.63)
					Pancreas	1	3.44 (0.04-19.18)
					Prostate	1	1.81 (0.02-10.11)
					Skin	4	0.80 (0.21-2.07)
					Stomach/esophagus	1	2.85 (0.03-15.89)
Björnådal et al. 2002[10]	Hospital Discharge Register	50,246 person-years	NA	5,715 (4,201 F/1,514 M)	All sites	443	1.25 (1.14-1.37)
					Bucca	11	1.50 (0.75-2.70)
					Digestive	111	1.14 (0.94-1.38)
					Esophagus	5	1.59 (0.52-3.71)
					Stomach	19	1.11 (0.67-1.73)
					Cardiac	3	1.97 (0.41-5.76)
					Large intestine	31	0.94 (0.63-1.35)
					Rectal	20	1.07 (0.66-1.66)
					Liver	17	1.61 (0.94-2.57)
					Pancreas	15	1.26 (0.70-2.07)
					Respiratory organs	50	1.79 (1.33-2.36)
					Larynx	6	3.42 (1.26-7.45)
					Lung	44	1.73 (1.25-2.32)
					Breast	52	0.72 (0.54-0.95)
					Female gen system	40	0.96 (0.69-1.31)
					Uterus	26	1.06 (0.70-1.56)
					Cervix	10	1.36 (0.65-2.51)
					Ovary	7	0.48 (0.19-0.99)
					Unspec fem	7	2.70 (1.09-5.57)
					Prostate	28	0.77 (0.51-1.11)
					Testis/other male cancer	1	1.14 (0.03-6.37)
					Kidney	11	0.97 (0.48-1.74)
					Bladder	23	1.32 (0.83-1.98)
					Melanoma	5	0.43 (0.14-1.01)
					Non melanoma skin	24	1.53 (0.98-2.28)
					Eye	1	1.07 (0.03-6.01)
					Brain	7	0.69 (0.28-1.41)
					Thyroid	1	0.29 (0.01-1.60)

					Endocrine	9	1.15 (0.52-2.18)
					Connective tissue	4	1.56 (0.42-3.98)
					Nos Cancer	19	1.35 (0.81-2.11)
					Hematologic	64	2.32 (1.78-2.96)
					HL	6	4.34 (1.59-9.45)
					NHL	32	2.86 (1.96-4.04)
					Multiple myeloma	7	1.19 (0.48-2.46)
					Leukemia	18	1.98 (1.18-3.13)
Bernatsky et al. 2003[11]	Montreal General Hospital and the Feinberg School of Medicine at Northwestern University	3,234 patient-years	ACR criteria	564 F	Breast	12	2.1 (1.1-3.7)
Ragnarsson et al. 2003[12]	Icelandic SLE database	2,774 patient-years 12.8 years	ARA criteria	238 (213 F/25 M)	All sites	27	1.38 (0.89-1.87)
					Breast	7	1.6 (0.65-3.23)
					Skin	3	6.43 (1.31-18.5)
					Lung	3	1.72 (0.36-4.95)
					Lymphoma	2	5.48 (0.64-19.6)
					Uterus	2	2.46 (0.29-8.78)
					Ovary	2	2.00 (0.23-7.14)
					Colon	1	0.88 (0.03-4.84)
					Larynx	1	23.60 (0.86-130)
					Vulva/vagina	1	10.44 (0.38-57.4)
					Thyroid	1	1.22 (0.06-9.40)
					Prostate	1	1.22 (0.03-6.17)
					Brain	1	1.81 (0.07-9.96)
					CLL	1	12.06 (0.44-66.3)
					CML	1	88.95 (2.37-450)
					Hematologic	4	8.89 (2.45-22.4)
Bernatsky et al. 2004[13]	SLE clinic cohorts at three centres	NA	ACR criteria	871 F	Breast	15	2.1 (1.1-3.5)
Bernatsky et al. 2005[14]	Multisite international SLE cohort	76,948 patient-years 8 years	ACR criteria	9,547 (8,592 F/955 M)	All sites	431	1.15 (1.05-1.27)
					Hematologic	67	2.75 (2.13-3.49)
					NHL	42	3.64 (2.63-4.93)
					HL	5	2.36 (0.75-5.51)
					Leukemia	7	1.89 (0.76-3.88)
					Breast	73	0.76 (0.60-0.95)
					Ovary	9	0.62 (0.28-1.18)

					Cervix	14	1.26 (0.69-2.11)
					Vagina	2	4.91 (0.49-17.69)
					Vulva	2	1.60 (0.16-5.76)
					Uterus	6	0.36 (0.13-0.78)
					Lung	62	1.37 (1.05-1.76)
					Hepatobiliary	10	2.60 (1.25-4.78)
					Pancreas	7	0.93 (0.37-1.91)
					Stomach	9	1.07 (0.49-2.03)
					Colorectum	40	1.01 (0.72-1.38)
					Thyroid	9	1.45 (0.66-2.76)
					Bladder	13	1.23 (0.66-2.11)
					Prostate	8	0.72 (0.31-1.43)
					Melanoma	9	0.97 (0.44-1.84)
Chun et al. 2005[15]	Hanyang Lupus Cohort	1,673 Person-years<br>4.7 years	ACR criteria	466<br>(434 F/32 M)	All sites	3	1.04 (0.21-3.03)
Bernatsky et al. 2005[16]	Multi-center SLE cohort	58,772 Person-years<br>10.0 years	ACR /clinical criteria	7,171	All sites	301	1.08 (0.91-1.30)[a]
					Lung	37	1.19 (0.84-1.67) [a]
					Breast	59	0.72 (0.55-0.93)
					Lymphoma	32	2.83 (1.94-3.99)
Tarr et al. 2007[17]	Hungarian lupus patients	14,190 patient-years<br>13.4 years	ACR criteria	860<br>(771 F/89 M)	All sites	37	0.89 (0.626-1.226)
		14,190 patient-years<br>11 years			Breast	11	0.62 (0.307-1.102)
		14,190 patient-years<br>23 years			Ovary	1	0.35 (0.005-1.945)
		14,190 patient-years<br>16.3 years			Cervix	5	1.74 (0.563-4.080)
		14,190 patient-years<br>8 years			Hematologic	5	1.31 (0.424-3.071)
		14,190 patient-years<br>8 years			NHL	2	3.47 (0.387-12.450)
		14,190 patient-years<br>7 years			Skin	1	0.04 (0.001-0.236)
		14,190 patient-years<br>8 years			Lung	4	0.48 (0.113-1.234)
		14,190 patient-years<br>25 years			Colorectum	5	0.52 (0.169-1.221)
		14,190 patient-years<br>23 years			Stomach	2	0.83 (0.094-3.009)
		14,190 patient-years<br>8.5 years			Oral	1	0.48 (0.006-2.688)
		14,190 patient-years			Bladder	1	0.34 (0.007-3.024)

		26 years<br>14,190 patient-years<br>6 years			Hepatobiliary	1	0.67 (0.009-3.709)
Parikh-Patel et al. 2008[18]	Statewide patient discharge data	157,969 person-years<br>5.1 years	ACR criteria	30,478<br>(27,133 F/3,345 M)	All sites	1,273	1.14 (1.07-1.20)
					Breast	237	0.76 (0.67-0.86)
					Ovary	27	0.82 (0.54-1.20)
					Uterus	29	0.6 (0.40-0.87)
					Vagina/vulva	49	3.27 (2.41-4.31)
					Cervix	38	0.55 (0.39-0.75)
					Prostate	43	0.69 (0.50-0.93)
					Lung	218	1.66 (1.45-1.90)
					Esophagus	12	1.85 (0.95-3.22)
					Stomach	18	1.23 (0.73-1.95)
					Colorectum	99	0.91 (0.74-1.11)
					Liver	17	2.7 (1.54-4.24)
					Pancreas	25	1.13 (0.73-1.67)
					Bladder	31	1.11 (0.75-1.57)
					Kidney	38	2.15 (1.52-2.94)
					Thyroid	30	1.83 (1.24-2.62)
					Br.1in/CNS	18	1.61 (0.95-2.54)
					NHL	96	2.74 (2.22-3.34)
					Large B-cell	42	3.26 (2.33-4.39)
					Follicular	26	2.89 (1.88-4.22)
					HL	13	3.02 (1.60-5.13)
					Leukemia	43	2.13 (1.49-2.77)
					Lymphoid	9	1.03 (0.47-1.96)
					Myeloid	29	2.96 (1.99-4.26)
					Monocytic	2	5 (0.68-20.17)
					Myeloma	15	1.35 (0.76-2.23)
					Melanoma	37	0.67 (0.47-0.93)
Kang et al. 2010[19]	Kangnam St. Mary's Hospital	5,716 person-years	ACR criteria	914 F	All sites	16	1.45 (0.74-2.16)
					Cervix	5	3.42 (0.00-7.26)
					NHL	3	15.37 (2.90-37.68)
					Bladder	3	43.55 (8.21-106.78)
					Uterus	1	3.94 (0.00-15.44)

					Ovary	1	2.62 (0.00-10.29)
					Lung	1	1.85 (0.00-7.26)
					Stomach	1	0.65 (0.00-2.54)
					Thyroid	1	0.98 (0.00-3.85)
Chen et al. 2010[20]	NHIRD	6.1 years	ARA criteria	11,763 (10,394 F/1,369 M)	All sites	259	1.76 (1.74-1.79)
					Hematologic	31	4.96 (4.79-5.14)
					Leukemia	7	2.64 (2.45-2.84)
					NHL	24	7.27 (6.98-7.57)
					Breast	45	1.55 (1.51-1.60)
					Uterus	5	1.28 (1.17-1.40)
					Cervix	22	1.39 (1.33-1.45)
					Ovary	3	0.72 (0.64-0.80)
					Prostate	2	0.79 (0.68-0.90)
					Vagina/vulva	3	4.76 (4.24-5.33)
					Skin	7	1.67 (1.55-1.80)
					Oropharynx/ larynx	9	2.03 (1.90-2.17)
					Hepatobiliary	28	1.83 (1.76-1.90)
					Colorectum	14	0.82 (0.78-0.86)
					Stomach	14	2.08 (1.97-2.19)
					Esophagus	2	1.63 (1.41-1.87)
					Pancreas	4	2.00 (1.81-2.21)
					Lung/mediastinum	16	1.23 (1.17-1.29)
					Bladder	2	0.66 (0.57-0.75)
					Nasopharynx,	10	4.18 (3.93-4.45)
					Kidney	9	3.99 (3.74-4.27)
					Brain	5	3.30 (3.00-3.59)
					Thyroid	14	2.24 (2.12-2.36)
					Others[h]	10	3.56 (3.34-3.79)
Liang et al. 2012[21]	NHIRD	16,789 person-years 8 years	NA	2,150 (1,664 F/486 M)	All sites	86	1.26 (0.99-1.59)
					Hematologic	NA	2.23 (0.95-5.24)
					Colorectum	NA	0.71 (0.30-1.65)
					Liver	NA	1.28 (0.66-2.47)
					Lung	18	1.41 (0.70-2.84)
					Breast	NA	0.78 (0.41-1.45)

					Uterus/cervical/ovary/vagina	NA	1.42 (0.66-3.03)
					Prostate	NA	3.78 (1.30-11.0)
Hemminki et al. 2012[22]	National MigMed 2 datasets at the Center for Primary Health Care Research	62,007 person-years	NA	5,318	Upper digestive tract	16	2.86 (1.63-4.65)
					Esophagus	2	1.52 (0.14-5.60)
					Stomach	10	1.20 (0.57-2.21)
					Colon	38	1.59 (1.13-2.19)
					Rectal	10	0.82 (0.39-1.51)
					Anus	7	7.18 (2.85-14.88)
Dreyer et al. 2011[23]	Danish Cancer Registry	7,803 person-years 13.2 years	ACR criteria	576 (508 F/68 M)	Virus-associated cancers	29	2.9 (2.0-4.1)
					HPV-associated cancers	19	2.3 (1.4-3.6)
					Oropharynx	1	1.8 (0.3-12.6)
					Anus	3	26.9 (8.7-83.4)
					Vagina/vulva	2	9.1 (2.3-36.5)
					Cervix	1	0.6 (0.1-4.5)
					Non-melanoma skin	12	2.0 (1.2-3.6)
					Other virus-associated cancers	10	4.3 (2.4-8.2)
					Liver	2	9.9 (2.5-39.8)
					NHL	4	5.0 (1.9-13.3)
					Bladder	4	3.6 (1.4-9.7)
					Esophagus	1	4.4 (0.6-31.3)
					Colon	4	1.8 (0.7-4.7)
					Rectum	1	0.9 (0.1-6.7)
					Pancreas	1	1.4 (0.2-9.9)
					Lung	5	1.4 (0.6-3.4)
					Breast	7	0.8 (0.4-1.6)
					Ovary	2	1.4 (0.3-5.4)
					Prostate	1	2.1 (0.3-15.0)
					Kidney	2	3.1 (0.8-12.4)
					Melanoma	2	1.3 (0.3-5.2)
					Brain	1	0.8 (0.1-5.4)
					Thyroid	1	3.5 (0.5-25.2)
					Leukemia	1	1.5 (0.2-10.9)
Hemminki et al. 2012[24]	National MigMed 2 datasets at the Center for Primary Health Care Research	86,640 person-years 11.9 years	NA	7,624	Lung	85	2.47 (1.97-3.05)

Lin et al. 2012[25]	NHIRD	NA	ARA criteria	9,349 F	Lymphoid	35	3.30 (2.20-4.93)
					Myeloid	14	2.86 (1.49-5.09)
Hemminki et al. 2012[26]	Swedish Hospital Discharge Register	86,627 person-years	NA	5,353	Breast	103	0.87 (0.71-1.06)
					Cervix	16	1.39 (0.79-2.27)
					Uterus	19	0.76 (0.46-1.19)
					Ovary	19	0.96 (0.58-1.51)
					Other female genital	10	2.97 (1.41-5.48)
Bernatsky et al. 2013[27]	Multi-center cohort	121,283 person-years 7.4 years	ACR criteria	16,409 (14,768 F/1,641 M)	All sites	644	1.14 (1.05-1.23)
					Hematologic	111	3.02 (2.48-3.63)
					NHL	76	4.39 (3.46-5.49)
					HL	7	2.28 (0.92-4.70)
					Multiple myeloma	10	1.88 (0.90-3.46)
					Leukemia	18	1.75 (1.04-2.76)
					Breast	114	0.73 (0.61-0.88)
					Ovary	13	0.64 (0.34-1.10)
					Cervix	21	1.27 (0.78-1.93)
					Vagina	2	3.80 (0.46-13.74)
					Vulva	7	3.78 (1.52-7.78)
					Uterus	12	0.44 (0.23-0.77)
					Lung	85	1.30 (1.04-1.60)
					Liver	12	1.87 (0.97-3.27)
					Pancreas	10	0.90 (0.43-1.65)
					Stomach	14	1.19 (0.65-2.00)
					Colorectum	51	0.88 (0.65-1.15)
					Thyroid	24	1.76 (1.13-2.61)
					Bladder	18	1.25 (0.74-1.97)
					Prostate	11	0.65 (0.32-1.16)
					Melanoma	11	0.67 (0.34-1.20)
Dey et al. 2013[28]	University College London Hospitals Lupus Clinic	8,910 person-years 14.7 years	ACR criteria	595	All sites	33	1.05 (0.52-1.58)
					Lung	5	1.72 (0.97-2.47)
					Breast	5	0.48 (0.35-0.64)
					Cervix	2	4.00 (3.50-4.50)
					Anus	2	1.80 (1.48-2.12)
					Prostate	3	4.29 (1.09-10.24)

					NHL	1	0.91 (0.87-0.95)
					Pancreas	1	1.43 (1.32-1.54)
Chang et al. 2013[29]	NHIRD	42,869 person-years	NA	8,751<br>(7,730 F/1,021 M)	All sites	151	1.56 (1.32-1.85)
					Head/neck	11	2.16 (1.13-4.13)
					Oral	5	2.05 (0.79-5.35)
					Oropharynx	1	6.06 (0.55-67.42)
					Salivary	1	3.22 (0.33-31.25)
					Nasopharynx	4	2.78 (0.92-8.39)
Hidalgo-Conde et al. 2013[30]	Hospital Universitario Virgen de la Victoria in Malaga	1,370 patient-years	ACR criteria	175<br>(158 F/17 M)	All sites	5	3.6 (1.5-8.6)
Bernatsky et al. 2013[31]	SLE registries at 10 pediatric centers	7,986 patient-years<br>7.8 years	NA	1,020<br>(836 F/184 M)	All sites	14	4.7 (2.6-7.8)
					Hematologic	3	5.2 (1.1-15.2)
Chan et al. 2016[32]	NHIRD	NA	ACR criteria	904<br>(774 F /130 M)	Lymphoma	6	22.82 (22.02-23.62)[b]
					Solid tumors	14	4.74 (4.21-5.27)[b]
Fallah et al. 2014[33]	Swedish health care databases	125,728<br>person-years<br>10.3 years	NA	12,207	NHL	107	4.4 (3.6-5.3)
Chang et al. 2014[34]	Seoul National University Hospital	10,410 person-years<br>8.9 years	NA	1,052<br>(935 F/117 M)	All sites	53	1.555 (1.137-1.974)
					Breast	4	0.68 (0.185-1.741)
					Cervix	7	4.282 (1.722-8.824)
					Colorectum	5	1.271 (0.413-2.967)
					NHL	5	7.408 (2.405-17.287)
					Stomach	3	0.597 (0.123-1.744)
					Thyroid	11	1.547 (0.633-2.461)
Khaliq et al. 2015[35]	Medicare claims dataset	5 years	NA	18,423 F	Breast	416	1.04 (0.90-1.21)
Yu et al. 2016[36]	NHIRD	124,832<br>person-years<br>35.3 years	NA	15,623<br>(13,693 F/1,930 M)	All sites	395	1.41 (1.28-1.56)
					Oral	NA	1.47 (0.81-2.65)
					Oropharynx/hypopharynx<br>/larynx	NA	1.23 (0.40-3.81)
					Nasopharynx	NA	2.3 (1.27-4.16)
					Esophagus	NA	0.83 (0.21-3.30)
					Stomach	NA	1.88 (1.21-2.91)
					Small intestine	NA	1.08 (0.15-7.63)
					Colorectum	NA	1.05 (0.75-1.45)
					Hepatobiliary	NA	1.5 (1.09-2.06)
					Biliary tract	NA	1.61 (0.60-4.29)
					Pancreas	NA	1.50 (0.68-3.35)

					Retroperitoneum/peritoneum	NA	3.73 (0.93-14.90)
					Lung	NA	1.38 (1.00-1.92)
					Bone/soft tissue sarcoma	NA	0.84 (0.21-3.36)
					Non melanoma skin cancer	NA	0.87 (0.39-1.93)
					Breast	NA	1.20 (0.96-1.51)
					Uterus	NA	0.31 (0.10-0.95)
					Cervix	NA	1.75 (1.22-2.52)
					Ovary	NA	0.93 (0.46-1.86)
					Prostate	NA	0.54 (0.14-2.16)
					Bladder	NA	1.08 (0.45-2.59)
					Kidney	NA	0.82 (0.21-3.29)
					Brain	NA	1.74 (0.65-4.64)
					Thyroid	NA	1.97 (1.35-2.87)
					NHL	NA	5.65 (4.02-7.95)
					Leukemia	NA	1.35 (0.65-2.84)
Goldman et al. 2016[37]	United States Kidney Data System	18,435 patient-years	NA	4,289	All sites	1622	3.5 (2.1-5.7)
					Lip/oropharyngeal	791	72 (57.3-92.0)
					Kaposi	38	38 (35.2-141.1)
					Neuroendocrine	163	32.6 (30.7-50.2)
					Thyroid	283	28.3 (16.2-41.2)
					Kidney	217	15.5 (12.3-21.7)
					Cervix	133	16.6 (3.0-27.3)
					Lymphoma	293	13.3 (9.0-14.6)
					Liver	40	6.6 (5.5-7.3)
					Colorectum	222	4.5 (3.2-6.1)
					Ovary	32	2.4 (1.3-5.6)
					Melanoma	27	1.4 (1.6-2.8)
					Breast	255	2.0 (1.1-3.1)
					Lung	43	0.7 (0.6-1.0)
					Prostate	54	0.33 (0.01-1.0)
Wadström et al. 2017[38]	Swedish national registers	25,666 person-years	ACR criteria	4,976 F	Cervix	5	1.64 (0.54-5.02)
Wang et al. 2018[39]	Guang An Men Hospital	NA	NA	225	All sites	2	0.605 (0.080-4.230)
					Lung	1	1.754 (0.158-6.450)
					Cervix	1	6.897 (2.748-14.144)

Bernatsky et al. 2017[40]	12 pediatric SLE registries in North America	NA	NA	1,168 (978 F/190 M)	Cervix	14	4.13 (2.26-6.93)
					NHL	3	4.68 (0.96-13.67)
Tallbacka et al. 2018[41]	Helsinki University Central Hospital	NA	ARA criteria	205 (182 F/23 M)	All sites	45	1.90 (1.39-2.54)
					Pharynx	1	13.5 (0.34-75.3)
					Gastrointestinal	7	1.57 (0.63-3.22)
					Stomach	1	1.20 (0.03-6.70)
					Colon	2	1.59 (0.19-5.73)
					Liver	1	4.42 (0.11-24.6)
					Pancreas	3	3.91 (0.81-11.4)
					Lung/trachea	3	2.20 (0.45-6.42)
					Melanoma	2	2.70 (0.33-9.75)
					Non melanoma skin	1	1.70 (0.04-9.45)
					Soft tissue sarcoma	2	12.1 (1.47-43.7)
					Breast	5	0.70 (0.23-1.63)
					Ovary	1	1.03 (0.03-5.74)
					Cervix	1	2.42 (0.06-13.47)
					Vagina/vulva	1	4.23 (0.11-23.54)
					Urinary system	7	6.34 (2.55-13.1)
					Kidney	5	7.79 (2.53-18.2)
					Bladder	2	4.33 (0.52-15.6)
					Brain	1	0.98 (0.02-5.47)
					Lymphatic/haematopoietic	12	6.40 (3.31-11.2)
					NHL	10	12.1 (5.82-22.3)
					Myeloma	1	3.38 (0.09-18.8)
Kuo et al. 2018[42]	Clinical Practice Research Data-link	NA	NA	1,605 (1,311 F/294 M)	Solid tumors /leukaemia	135	1.29 (1.04-1.61)
					Lymphoma	21	3.19 (1.54-6.64)
					Metastatic solid tumours	24	2.31 (1.33-4.00)
Bae et al. 2019[43]	Korean National Health Insurance Service database	1,000 person-years	NA	21,016 (18,960 F/2,056 M)	All sites	763	1.446 (1.331-1.568)
					Stomach	NA	0.794 (0.555-1.107)
					Colorectum	NA	1.243 (0.979-1.563)
					Liver	NA	1.419 (0.965-2.033)
					Pancreas	NA	0.930 (0.543-1.503)
					Lung	NA	1.336 (0.936-1.865)
					Breast	NA	0.953 (0.766-1.174)

					Cervix	NA	3.085 (2.298-4.115)
					Thyroid	NA	1.304 (1.128-1.503)
					Lymphoma	NA	6.274 (4.106-9.635)
					Ovary	NA	1.710 (1.121-2.546)
					Prostate	NA	1.103 (0.474-2.270)
					Oral	NA	2.867 (1.553-5.098)
					Biliary	NA	0.828 (0.381-1.60)
					Larynx	NA	3.254 (0.403-20.718)
					Kidney	NA	1.330 (0.671-2.467)
					Bladder	NA	1.912 (0.876-3.846)
					Nerve	NA	1.147 (0.493-2.356)
					Multiple myeloma	NA	3.825 (1.541-9.149)
					Leukemia	NA	2.504 (1.235-4.824)
					Skin	NA	2.405 (0.649-7.426)
Wang et al. 2019[44]	NHIRD	NA	ACR criteria	16,417 (14,293 F/2,124 M)	All sites	512	1.37 (1.26-1.50)
					NHL	34	4.2 (2.9-5.9)
Cobo-Ibáñez et al. 2020[45]	SLE Registry of the Spanish Society of Rheumatology	NA	ACR criteria	3,539 (3,194 F/345 M)	All sites	154	1.37 (1.15-1.59)

Cancer-specific mortality in SLE patients

Author	Sources of SLE patients	Follow-up (person-years average years)	SLE diagnosis	Number of SLE patients (gender)	Cancer site	Death	SMR (95% CI)
Nived et al. 2001[46]	Health Care Districts of Lund and Orup	1,086 patient-years 9.4 years	ACR criteria	116	All sites	11	1.37 (0.65-2.90)[c]
					NHL	2	11.63 (1.4-42.0)
					Lung	2	5.55 (0.7-20.1)
					Prostate	3	6.41 (1.3-18.7)
Bernatsky et al. 2006[47]	Multisite international SLE cohort	76,948 patient-years 8.1 years	ACR criteria	9,547 (8,607 F/940 M)	All sites	114	0.8 (0.6-1.0)
					Hematologic	15	2.1 (1.2-3.4)
					NHL	8	2.8 (1.2-5.6)
					Lung	44	2.3 (1.6-3.0)
Hemminki et al. 2012[24]	National MigMed 2 datasets at the Center for Primary Health Care Research	91,669 person-years 11.9 years	NA	7,624	Lung	73	2.69 (2.11-3.38)
Hemminki et al. 2012[26]	Swedish Hospital Discharge Register		NA	5,353	All sites	42	1.19 (0.88-1.62)[d]
		661 person-years			Breast	23	1.21 (0.81-1.82)
		191 person-years			Cervix	1	0.25 (0.04-1.75)

		143 person-years			Uterus	3	2.14 (0.69-6.64)
		89 person-years			Ovary	12	1.07 (0.61-1.88)
		48 person-years			Other female genital	3	1.61 (0.52-5.00)
Lerang et al. 2014[48]	Multiple sources	NA	ACR criteria	325<br>(291 F/34 M)	All sites	NA	2.23 (1.02-4.90)[e]
Tselios et al. 2019[49]	University of Toronto Lupus Clinic	NA	ACR criteria	1,732	All sites	24	3.4 (2.2-4.6)
Bultink et al. 2021[50]	Clinical Practice Research Datalink	NA	NA	4,356<br>(3,878 F/478 M)	All sites	95	1.90 (1.50-2.40)
				2,603	All sites	88	0.65 (0.47-0.90)
					Solid tumours	73	0.56 (0.38-0.80)
					Hematologic	26	0.64 (0.35-1.17)

a. The RR was a pooled OR based on ORs for different ethnicity in the original paper

b. The RR was calculated by 2 × 2 table

c. The RR was a pooled RR based on RRs for male and female.

d. The RR was a pooled RR based on RRs for different type of cancer in the original paper

e. The RR was a pooled RR based on RRs for different age in the original paper

f. Two cases of acute myeloid leukemia and 1 case of chronic lymphocytic leukemia

g. Hodgkin's disease (One adenocarcinoma (unspecified site), 1 adenosquamous carcinoma (peritoneum), 1 squamous cell carcinoma (head and neck), and 1 carcinoma not otherwise specified (brain).

h. malignancies of salivary glands, intestine, retroperitoneum, bone, cartilage, and connective tissue

ACR: American College of Rheumatology, ARA: American Rheumatism Association, CI: confidence intervals, CLL: Chronic lymphocytic leukaemia, CML: Chronic myeloid leukaemia, F: female, HL: Hodgkin's lymphoma, M: Male, NA: Not available, NHIRD: National Health Insurance Research Database, NHL: non-Hodgkinb Lymphoma, RR: Relative risk, SLE: systemic lupus erythematosus, SMR: standard mortality ratio.

Supplementary Table S2. Quality of the included studies, according to the Newcastle–Ottawa Scale.

Study	Selection	Comparability	Exposure/ Outcome	Total number of stars
Pettersson et al. 1992[3]	★★★	★★	★★	7
Sweeney et al. 1995[4]	★★★	★	★★	6
Abu-Shakra et al. 1996[5]	★★★★	★	★★	7
Mellemkjaer et al. 1997[6]	★★★★	★	★★★	8
Sultan et al. 2000[7]	★★★★	★★	★★★	9
Goldman et al. 1998[8]	★★★	★	★★	6
Cibere et al. 2001[8]	★★★★	★★	★★★	9
Nived et al. 2001[46]	★★★★	★★	★★★	9
Björnådal et al. 2002[10]	★★★★	★★	★★	8
Bernatsky et al. 2003[11]	★★★★	★★	★★★	9
Ragnarsson et al. 2003[12]	★★★★	★	★★★	8
Bernatsky et al. 2004[13]	★★★★	★★	★★★	9
Bernatsky et al. 2005[14]	★★★★	★★	★★★	9
Chun et al. 2005[15]	★★★	★	★★	6
Bernatsky et al. 2005[16]	★★★★	★★	★★	8
Bernatsky et al. 2006[47]	★★★★	★★	★★★	9
Tarr et al. 2007[17]	★★★★	★★	★★	8
Parikh-Patel et al. 2008[18]	★★★★	★★	★★★	9
Kang et al. 2010[19]	★★★★	★★	★★	8
Chen et al. 2010[20]	★★★★	★	★★	7
Liang et al. 2012[21]	★★★★	★★	★★★	9
Hemminki et al. 2012[22]	★★★★	★★	★★	8
Dreyer et al. 2011[23]	★★★★	★	★★	7
Hemminki et al. 2012[24]	★★★★	★	★★	7
Lin et al. 2012[25]	★★★★	★★	★★	8
Hemminki et al. 2012[26]	★★★	★★	★★	7
Bernatsky et al. 2013[27]	★★★★	★★	★★	8
Dey et al. 2013[28]	★★★★	★★	★★★	9
Chang et al. 2013[29]	★★★★	★★	★★★	9
Hidalgo-Conde et al. 2013[30]	★★★★	★★	★★★	9
Bernatsky et al. 2013[31]	★★★	★★	★★	7
Chan et al. 2016[32]	★★★★	★	★★★	8
Fallah et al. 2014[33]	★★★★	★	★★	7
Chang et al. 2014[34]	★★★★	★★	★★★	9
Lerang et al. 2014	★★★★	★★	★★★	9
Khaliq et al. 2015[35]	★★★	★★	★★	7
Yu et al. 2016[36]	★★★★	★	★★★	8
Goldman, et al. 2016[37]	★★★	★★	★★	7
Wadström et al. 2017[38]	★★★	★★	★★★	8
Wang et al. 2018[39]	★★★	★	★★	6

Bernatsky et al. 2017[40]	★★★★	★★	★★	8
Tallbacka et al. 2018[41]	★★★	★	★★	6
Kuo et al. 2018[42]	★★★★	★★	★★	8
Bae et al. 2019[43]	★★★★	★★	★★	8
Wang et al. 2019[44]	★★★★	★★	★★	8
Tselios et al. 2019[49]	★★★★	★★	★★	8
Cobo-Ibáñez et al. 2020[45]	★★★★	★	★★	7
Bultink et al. 2021[50]	★★★★	★★	★★	8

Supplementary Table S3. SNPs associated with systemic lupus erythematosus identified by the largest published Genome-wide association studies (GWASs) in European ancestry

SNP	Effect allele	Other allele	Chr	Position	MAF	Beta	SE	P value	N
rs74290525	G	A	6	31867385	0.02	0.723	0.101	1.00E-12	23210
rs12802200	C	A	11	566936	0.17	0.207	0.034	9.00E-10	23210
rs13332649	A	G	16	85933077	0.20	0.293	0.033	2.00E-18	23210
rs11644034	G	A	16	85939006	0.20	0.223	0.026	1.00E-17	23210
rs887369	C	A	X	30559729	0.24	0.14	0.022	5.00E-10	23210
rs268134	G	A	2	65381229	0.25	0.191	0.029	1.00E-10	23210
rs9782955	C	T	1	235876577	0.25	0.148	0.024	1.00E-09	23210
rs58688157	A	G	11	625085	0.27	0.215	0.03	5.00E-13	23210
rs17849501	T	G	1	183573188	0.28	0.742	0.038	3.00E-88	23210
rs2304256	C	A	19	10364976	0.29	0.215	0.03	4.00E-13	23210
rs9652601	G	A	16	11080508	0.29	0.191	0.023	7.00E-17	23210
rs11085727	C	T	19	10355447	0.30	0.215	0.029	1.00E-13	23210
rs4917014	T	G	7	50266267	0.32	0.166	0.022	6.00E-14	23210
rs7097397	G	A	10	48817351	0.34	0.182	0.027	9.00E-12	23210
rs10028805	G	A	4	101816093	0.34	0.182	0.022	4.00E-17	23210
rs10753074	T	C	1	173377204	0.38	0.191	0.028	6.00E-12	23210
rs564799	C	T	3	160011200	0.39	0.131	0.022	2.00E-09	23210
rs2111485	G	A	2	162254026	0.39	0.14	0.021	1.00E-11	23210
rs2732549	A	G	11	35066852	0.39	0.215	0.021	1.00E-23	23210
rs9311676	C	T	3	58484624	0.40	0.157	0.021	3.00E-14	23210
rs2431697	T	C	5	160452971	0.46	0.231	0.021	8.00E-28	23210
rs7941765	C	T	11	128629105	0.48	0.131	0.02	1.00E-10	23210
rs12531540	C	T	7	28123055	0.49	0.14	0.025	3.00E-08	23210
rs849142	T	C	7	28146272	0.49	0.131	0.02	9.00E-11	23210
rs6671847	A	G	1	161509020	0.47	0.182	0.026	1.00E-12	23210
rs10774625	A	G	12	111472415	0.47	0.122	0.021	4.00E-09	23210
rs4948496	C	T	10	62045858	0.47	0.131	0.02	1.00E-10	23210
rs6568431	A	C	6	106140931	0.46	0.191	0.025	5.00E-14	23210
rs597808	A	G	12	111535554	0.45	0.166	0.027	6.00E-10	23210
rs2431098	G	A	5	160460329	0.45	0.223	0.026	4.00E-18	23210
rs6736175	C	T	2	191081596	0.42	0.215	0.026	9.00E-17	23210
rs3757387	C	T	7	128936032	0.42	0.372	0.025	1.00E-48	23210
rs4902562	A	G	14	68264741	0.41	0.131	0.021	6.00E-10	23210
rs1059312	G	A	12	128794319	0.40	0.157	0.021	1.00E-13	23210
rs11059919	G	A	12	128804645	0.39	0.148	0.026	8.00E-09	23210
rs10912578	A	G	1	173282717	0.31	0.239	0.027	4.00E-19	23210
rs3794060	C	T	11	71476633	0.30	0.207	0.022	1.00E-20	23210
rs704840	G	T	1	173257056	0.28	0.199	0.022	3.00E-19	23210
rs2289583	A	C	15	75018695	0.28	0.174	0.022	6.00E-15	23210
rs9462027	A	G	6	34829464	0.26	0.131	0.023	8.00E-09	23210
rs2736332	C	G	8	11482456	0.26	0.27	0.028	2.00E-22	23210
rs2736340	T	C	8	11486464	0.24	0.255	0.028	6.00E-20	23210
rs11889341	T	C	2	191079016	0.22	0.548	0.024	6.00E-122	23210
rs10036748	T	C	5	151078585	0.21	0.322	0.024	1.00E-45	23210

rs9273076	T	C	6	32644524	0.20	0.262	0.037	8.00E-13	23210
rs3024505	A	G	1	206766559	0.19	0.157	0.027	5.00E-09	23210
rs3747093	A	G	22	21630090	0.19	0.231	0.032	3.00E-13	23210
rs820077	G	A	6	35066077	0.18	0.174	0.03	1.00E-08	23210
rs7444	C	T	22	21622645	0.18	0.239	0.025	2.00E-22	23210
rs1734787	C	A	X	154059995	0.17	0.27	0.034	2.00E-15	23210
rs114090659	C	T	6	30973212	0.16	0.688	0.034	6.00E-92	23210
rs3957147	T	C	6	32714358	0.13	0.693	0.034	3.00E-93	23210
rs2476601	A	G	1	113834946	0.12	0.358	0.032	1.00E-28	23210
rs35472514	G	C	16	31272002	0.12	0.531	0.035	4.00E-53	23210
rs35000415	T	C	7	128945562	0.11	0.604	0.037	1.00E-60	23210
rs10488631	C	T	7	128954129	0.11	0.652	0.029	9.00E-110	23210
rs34572943	A	G	16	31261032	0.10	0.536	0.03	3.00E-76	23210
rs10048743	G	T	2	213025508	0.09	0.223	0.035	2.00E-10	23210
rs3768792	G	A	2	213006985	0.09	0.215	0.029	1.00E-13	23210
rs1150757	A	G	6	32061428	0.09	0.846	0.039	6.00E-107	23210
rs1270942	G	A	6	31951083	0.08	0.824	0.03	2.00E-165	23210
rs2286672	T	C	17	4809322	0.08	0.223	0.038	3.00E-09	23210
rs2941509	T	C	17	39764941	0.03	0.3	0.052	8.00E-09	23210
rs4388254	T	C	5	134092910	0.03	0.336	0.055	7.00E-10	23210
rs7726414	T	C	5	134096143	0.03	0.372	0.046	4.00E-16	23210
rs58721818	T	C	6	137922602	0.03	0.599	0.067	3.00E-19	23210
rs143123127	A	G	17	39850937	0.03	0.412	0.071	6.00E-09	23210
rs6932056	C	T	6	137921300	0.02	0.604	0.052	2.00E-31	23210
rs77583790	A	G	3	159976265	0.01	0.765	0.117	6.00E-11	23210

Chr: Chromosome, MAF: Minor allele frequency, N: Simple size, SNP: Single nucleotide polymorphism.

Supplementary Table S4. Details of outcome included in Mendelian randomization analyses

Trait	First author	Year	Consortium	Number of cases	Number of controls	Sample size
Colon cancer	Neale lab	2018	NA	2,226	358,968	361,194
Pancreatic cancer	NA	2020	NA	229	96,270	96,499
Lung cancer	Wang Y	2014	ILCCO	11,348	15,861	27,209
Cervical cancer	Ben Elsworth	2018	MRC-IEU	3,175	459,835	463,010
Bladder cancer	Neale lab	2018	NA	1,554	359,640	361,194
Lymphoma	Neale lab	2018	NA	1,752	359,442	361,194
Non-melanoma skin cancer	Neale	2017	Neale Lab	4,826	332,373	337,199

Supplementary Table S5. **Mendelian randomization estimates between systemic lupus erythematosus and cancer risk in European ancestry.**

Outcome	IVW		MR Egger		weighted median		weighted mode		Horizontal pleiotropy	Q_pval
	OR (95% CI)	P	OR (95% CI)	P	OR (95% CI)	P	OR (95% CI)	P		
Colon cancer	1.0000 (0.9998-1.0002)	0.92	1.0003 (0.9998-1.0007)	0.22	1.0002 (0.9999-1.0005)	0.28	1.0003 (0.9998-1.0007)	0.22	0.16	0.16
Pancreatic cancer	1.0169 (0.9123-1.1335)	0.76	1.0075 (0.8066-1.2584)	0.95	1.0022 (0.8529-1.1777)	0.98	1.0238 (0.8159-1.2848)	0.84	0.92	0.48
Lung cancer	1.0239 (0.9934-1.0555)	0.13	1.0802 (1.0156-1.1489)	0.01	1.0334 (0.9966-1.0716)	0.08	1.0699 (0.9202-1.2439)	0.38	0.048	8E-07
Cervical cancer	1.0001 (0.9999-1.0002)	0.53	0.9996 (0.9993-1.0000)	0.048	0.9999 (0.9996-1.0002)	0.47	0.9999 (0.9995-1.0002)	0.41	0.008	0.87
Bladder cancer	**0.9996 (0.9994-0.9998)**	**4E-05**	0.9994 (0.9991-0.9998)	0.002	0.9996 (0.9994-0.9999)	0.007	0.9996 (0.9993-1.0000)	0.03	0.22	0.76
Lymphoma	**1.0004 (1.0001-1.0007)**	**0.0035**	1.0008 (1.0003-1.0013)	0.004	1.0003 (1.0000-1.0006)	0.07	1.0002 (0.9996-1.0008)	0.44	0.09	1E-07
Non-melanoma skin cancer	0.9997 (0.9996-1.0003)	0.89	1.0008 (1.0001-1.0015)	0.02	1.0001 (0.9996-1.0006)	0.69	1.0005 (0.9998-1.0011)	0.15	0.006	0.11

IVW: inverse variance weighted.

Supplementary Figure S1. Funnel plots of MR estimate concerning relationship between SLE and lymphoma. The causal effect of exposure on outcome is estimated using each SNP singly using the Wald ratio.

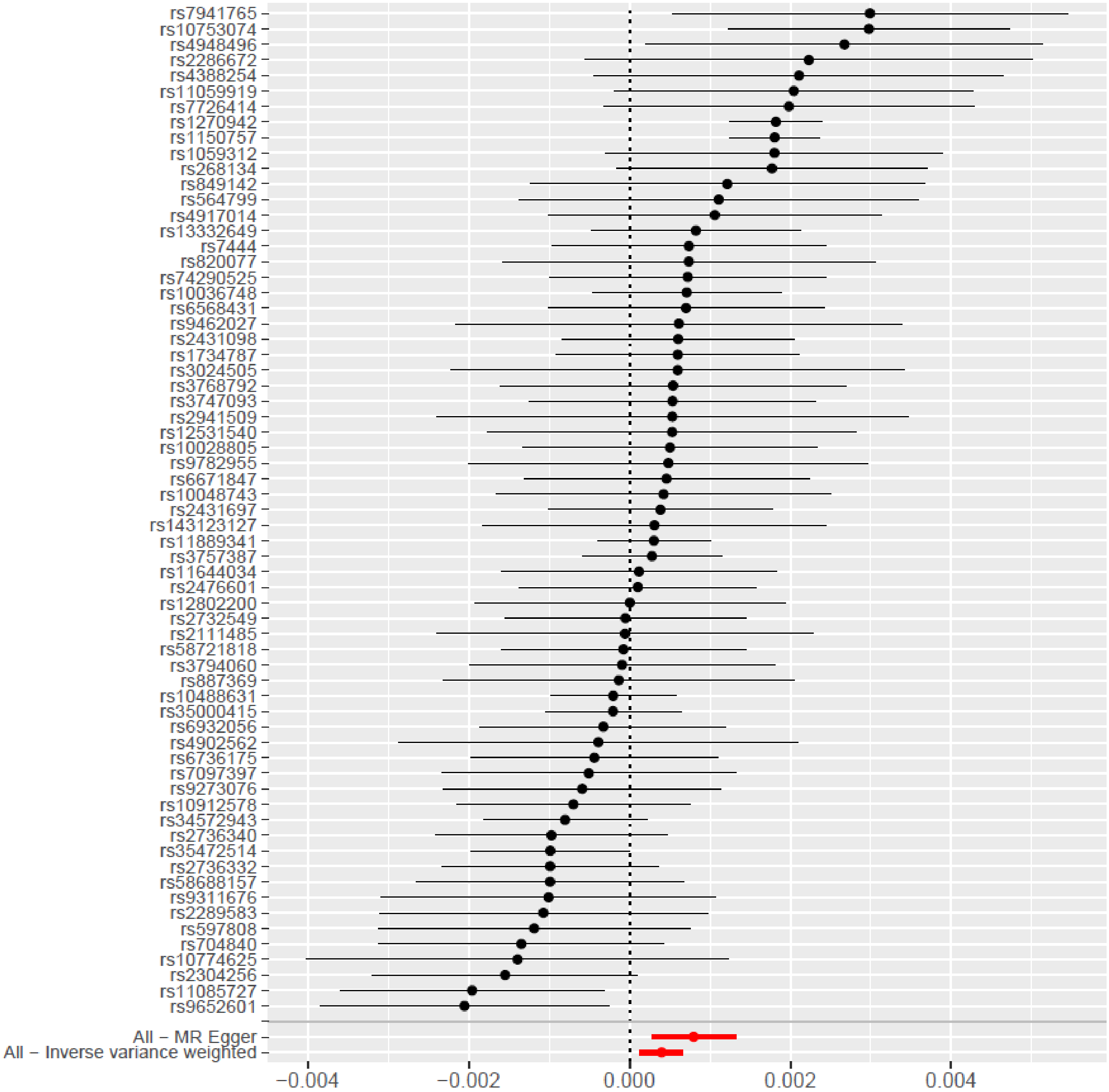

Supplementary Figure S2. Scatter plots of MR estimate concerning relationship between SLE and lymphoma. SNP effects on the outcome are plotted against SNP effects on the exposure, and the slope of the line represents the causal association.

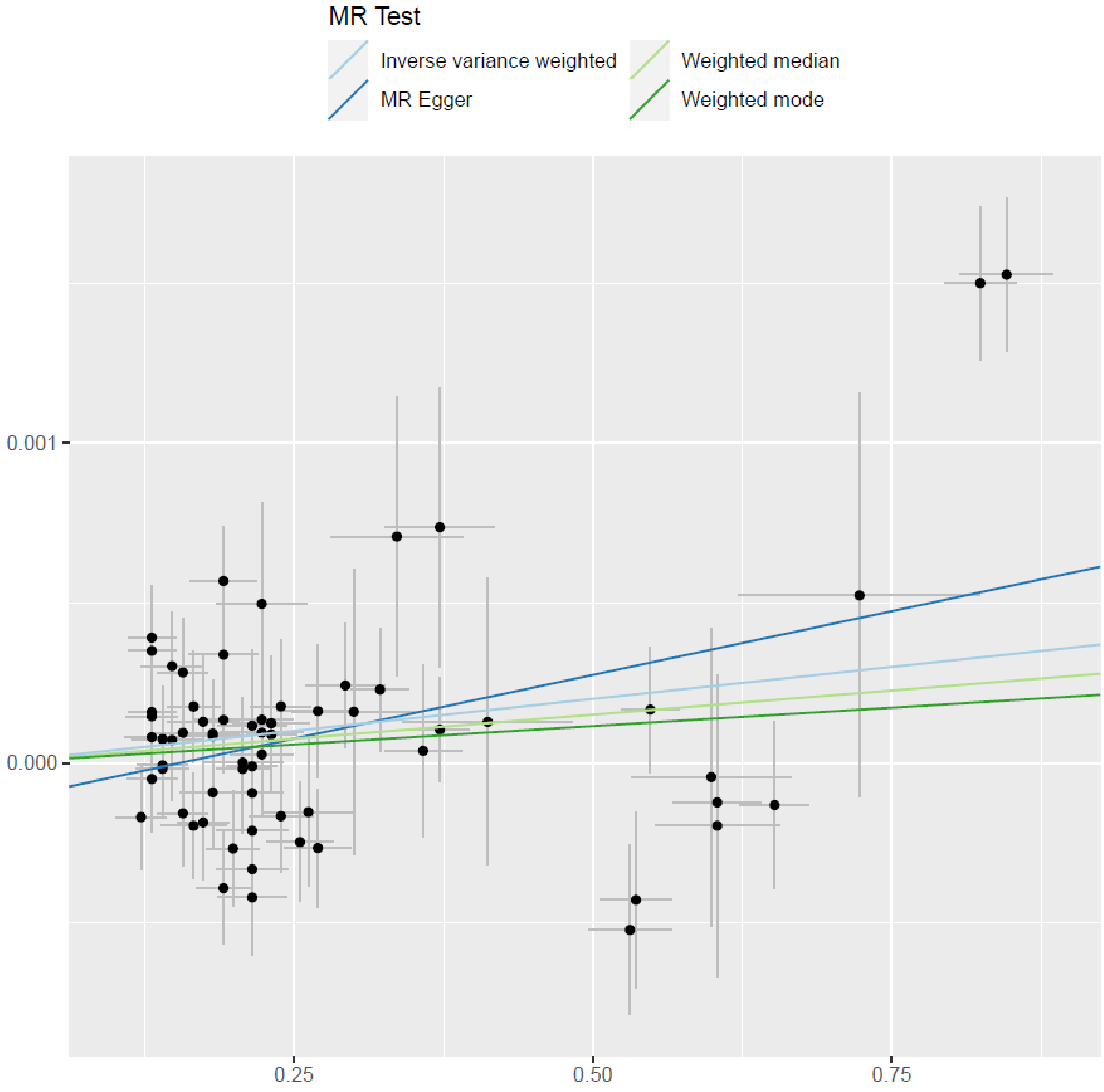

Supplementary reference

1. Bentham J., Morris D.L., Graham D.S.C., Pinder C.L., Tombleson P., Behrens T.W., et al., Genetic association analyses implicate aberrant regulation of innate and adaptive immunity genes in the pathogenesis of systemic lupus erythematosus. *Nat Genet* (2015) 47(12): 1457-1464. doi: 10.1038/ng.3434.
2. Yarmolinsky J., Bonilla C., Haycock P.C., Langdon R.J.Q., Lotta L.A., Langenberg C., et al., Circulating Selenium and Prostate Cancer Risk: A Mendelian Randomization Analysis. *J Natl Cancer Inst* (2018) 110(9): 1035-1038. doi: 10.1093/jnci/djy081.
3. Pettersson T., Pukkala E., Teppo L., and Friman C., Increased risk of cancer in patients with systemic lupus erythematosus. *Ann. Rheum. Dis* (1992) 51(4): 437-439. doi: 10.1136/ard.51.4.437.
4. Sweeney D.M., Manzi S., Janosky J., Selvaggi K.J., Ferri W., Medsger T.A., Jr., et al., Risk of malignancy in women with systemic lupus erythematosus. *J. Rheumatol* (1995) 22(8): 1478-1482. doi.
5. Abu-Shakra M., Gladman D.D., and Urowitz M.B., Malignancy in systemic lupus erythematosus. *Arthritis Rheum* (1996) 39(6): 1050-1054. doi: 10.1002/art.1780390625.
6. Mellemkjaer L., Andersen V., Linet M.S., Gridley G., Hoover R., and Olsen J.H., Non-Hodgkin's lymphoma and other cancers among a cohort of patients with systemic lupus erythematosus. *Arthritis Rheum* (1997) 40(4): 761-768. doi: 10.1002/art.1780400424.
7. Sultan S.M., Ioannou Y., and Isenberg D.A., Is there an association of malignancy with systemic lupus erythematosus? An analysis of 276 patients under long-term review. *Rheumatology (Oxford)* (2000) 39(10): 1147-52. doi: 10.1093/rheumatology/39.10.1147.
8. Ramsey-Goldman R., Mattai S.A., Schilling E., Chiu Y.L., Alo C.J., Howe H.L., et al., Increased risk of malignancy in patients with systemic lupus erythematosus. *J. Investig. Med* (1998) 46(5): 217-222.
9. Cibere J., Sibley J., and Haga M., Systemic lupus erythematosus and the risk of malignancy. *Lupus* (2001) 10(6): 394-400. doi: 10.1191/096120301678646128.
10. Bjrndal L., Lfstrm B., Yin L., Lundberg I.E., and Ekbom A., Increased cancer incidence in a Swedish cohort of patients with systemic lupus erythematosus. *Scand. J. Rheumatol* (2002) 31(2): 66-71. doi: 10.1080/03009740252937568.
11. Bernatsky S., Ramsey-Goldman R., Boivin J.F., Joseph L., Moore A.D., Rajan R., et al., Do traditional Gail model risk factors account for increased breast cancer in women with lupus? *J. Rheumatol* (2003) 30(7): 1505-1507. doi: 0315162X-30-1505.
12. Ragnarsson O., Grndal G., and Steinsson K., Risk of malignancy in an unselected cohort of Icelandic patients with systemic lupus erythematosus. *Lupus* (2003) 12(9): 687-691. doi: 10.1191/0961203303lu443oa [doi].
13. Bernatsky S., Clarke A., Ramsey-Goldman R., Joseph L., Boivin J.F., Rajan R., et al., Hormonal exposures and breast cancer in a sample of women with systemic lupus erythematosus. *Rheumatology. (Oxford)* (2004) 43(9): 1178-1181. doi: keh282 [pii];10.1093/rheumatology/keh282.
14. Bernatsky S., Boivin J.F., Joseph L., Rajan R., Zoma A., Manzi S., et al., An international cohort study of cancer in systemic lupus erythematosus. *Arthritis Rheum* (2005) 52(5): 1481-1490. doi: 10.1002/art.21029.
15. Chun B.C. and Bae S.C., Mortality and cancer incidence in Korean patients with systemic lupus erythematosus: results from the Hanyang lupus cohort in Seoul, Korea. *Lupus* (2005) 14(8): 635-638. doi: 10.1191/0961203305lu2180xx [doi].
16. Bernatsky S., Boivin J.F., Joseph L., Manzi S., Ginzler E., Urowitz M., et al., Race/ethnicity and cancer occurrence in systemic lupus erythematosus. *Arthritis Rheum* (2005) 53(5): 781-784. doi: 10.1002/art.21458.
17. Tarr T., Gyorfy B., Szekanecz E., Bhattoa H.P., Zeher M., Szegedi G., et al., Occurrence of malignancies in Hungarian patients with systemic lupus erythematosus: results from a single center. *Ann. N. Y. Acad. Sci* (2007) 1108: 76-82. doi: 10.1196/annals.1422.008.
18. Parikh-Patel A., White R.H., Allen M., and Cress R., Cancer risk in a cohort of patients with systemic lupus erythematosus (SLE) in California. *Cancer Causes Control* (2008) 19(8): 887-894. doi: 10.1007/s10552-008-9151-8.
19. Kang K.Y., Kim H.O., Yoon H.S., Lee J., Lee W.C., Ko H.J., et al., Incidence of cancer among female patients with systemic lupus erythematosus in Korea. *Clin. Rheumatol* (2010) 29(4): 381-388. doi: 10.1007/s10067-009-1332-7.
20. Chen Y.J., Chang Y.T., Wang C.B., and Wu C.Y., Malignancy in systemic lupus erythematosus: a nationwide cohort study in Taiwan. *Am. J. Med* (2010) 123(12): 1150-1156. doi: S0002-9343(10)00744-8 [pii];10.1016/j.amjmed.2010.08.006.
21. Liang J.A., Sun L.M., Yeh J.J., Lin W.Y., Chang S.N., Sung H.C., et al., Malignancies associated with systemic lupus

erythematosus in Taiwan: a nationwide population-based cohort study. *Rheumatol. Int* (2012) 32(3): 773-778. doi: 10.1007/s00296-010-1684-y.

22. Hemminki K., Liu X., Ji J., Sundquist J., and Sundquist K., Autoimmune disease and subsequent digestive tract cancer by histology. *Ann. Oncol* (2012) 23(4): 927-933. doi: S0923-7534(19)34642-3 [pii];10.1093/annonc/mdr333.
23. Dreyer L., Faurschou M., Mogensen M., and Jacobsen S., High incidence of potentially virus-induced malignancies in systemic lupus erythematosus: a long-term followup study in a Danish cohort. *Arthritis Rheum* (2011) 63(10): 3032-3037. doi: 10.1002/art.30483.
24. Hemminki K., Liu X., Ji J., Sundquist J., and Sundquist K., Effect of autoimmune diseases on risk and survival in histology-specific lung cancer. *Eur. Respir. J* (2012) 40(6): 1489-1495. doi: 09031936.00222911 [pii];10.1183/09031936.00222911.
25. Lin Y.C., Yen J.H., Chang S.J., and Lin Y.C., The age-risk relationship of haematologic malignancies in female patients with systemic lupus erythematosus: a nationwide retrospective cohort study. *Lupus* (2012) 21(11): 1250-1256. doi: 0961203312451783 [pii];10.1177/0961203312451783.
26. Hemminki K., Liu X., Ji J., Frsti A., Sundquist J., and Sundquist K., Effect of autoimmune diseases on risk and survival in female cancers. *Gynecol. Oncol* (2012) 127(1): 180-185. doi: S0090-8258(12)00621-X [pii];10.1016/j.ygyno.2012.07.100.
27. Bernatsky S., Ramsey-Goldman R., Labrecque J., Joseph L., Boivin J.F., Petri M., et al., Cancer risk in systemic lupus: an updated international multi-centre cohort study. *J Autoimmun* (2013) 42: 130-5. doi: 10.1016/j.jaut.2012.12.009.
28. Dey D., Kenu E., and Isenberg D.A., Cancer complicating systemic lupus erythematosus--a dichotomy emerging from a nested case-control study. *Lupus* (2013) 22(9): 919-927. doi: 0961203313497118 [pii];10.1177_0961203313497118 [pii];10.1177/0961203313497118.
29. Chang S.L., Hsu H.T., Weng S.F., and Lin Y.S., Impact of head and neck malignancies on risk factors and survival in systemic lupus erythematosus. *Acta Otolaryngol* (2013) 133(10): 1088-1095. doi: 10.3109/00016489.2013.800228 [doi].
30. Hidalgo-Conde A., de Haro L.M., Abarca-Costalago M., lvarez P.M., Valdivielso-Felices P., Gonzlez-Santos P., et al., Incidence of cancer in a cohort of Spanish patients with systemic lupus erythematosus. *Reumatol. Clin* (2013) 9(6): 359-364. doi: S1699-258X(13)00012-0 [pii];10.1016/j.reuma.2012.10.015.
31. Bernatsky S., Clarke A.E., Labrecque J., von S.E., Schanberg L.E., Silverman E.D., et al., Cancer risk in childhood-onset systemic lupus. *Arthritis Res. Ther* (2013) 15(6): R198. doi: ar4388 [pii];10.1186/ar4388.
32. Chan P.C., Yu C.H., Yeh K.W., Horng J.T., and Huang J.L., Comorbidities of pediatric systemic lupus erythematosus: A 6-year nationwide population-based study. *J. Microbiol. Immunol. Infect* (2016) 49(2): 257-263. doi: S1684-1182(14)00097-8 [pii];10.1016/j.jmii.2014.05.001.
33. Fallah M., Liu X., Ji J., Fersti A., Sundquist K., and Hemminki K., Autoimmune diseases associated with non-Hodgkin lymphoma: a nationwide cohort study. *Ann. Oncol* (2014) 25(10): 2025-2030. doi: S0923-7534(19)36624-4 [pii];10.1093/annonc/mdu365].
34. Chang S.H., Park J.K., Lee Y.J., Yang J.A., Lee E.Y., Song Y.W., et al., Comparison of cancer incidence among patients with rheumatic disease: a retrospective cohort study. *Arthritis Res. Ther* (2014) 16(4): 428. doi: s13075-014-0428-x [pii];428 [pii];10.1186/s13075-014-0428-x.
35. Khaliq W., Qayyum R., Clough J., Vaidya D., Wolff A.C., and Becker D.M., Comparison of breast cancer risk in women with and without systemic lupus erythematosus in a Medicare population. *Breast Cancer Res. Treat* (2015) 151(2): 465-474. doi: 10.1007/s10549-015-3412-5.
36. Yu K.H., Kuo C.F., Huang L.H., Huang W.K., and See L.C., Cancer Risk in Patients With Inflammatory Systemic Autoimmune Rheumatic Diseases: A Nationwide Population-Based Dynamic Cohort Study in Taiwan. *Medicine (Baltimore)* (2016) 95(18): e3540. doi: 00005792-201605030-00026 [pii];10.1097/MD.0000000000003540.
37. Ramsey-Goldman R., Brar A., Richardson C., Salifu M.O., Clarke A., Bernatsky S., et al., Standardised incidence ratios (SIRs) for cancer after renal transplant in systemic lupus erythematosus (SLE) and non-SLE recipients. *Lupus Sci. Med* (2016) 3(1): e000156. doi: lupus-2016-000156 [pii];10.1136/lupus-2016-000156.
38. Wadstrem H., Arkema E.V., Sjewall C., Askling J., and Simard J.F., Cervical neoplasia in systemic lupus erythematosus: a nationwide study. *Rheumatology. (Oxford)* (2017) 56(4): 613-619. doi: kew459 [pii];10.1093/rheumatology/kew459.
39. Wang H.L., Zhou Y.M., Zhu G.Z., Yang Z., and Hua B.J., Malignancy as a comorbidity in rheumatic diseases: a retrospective hospital-based study. *Clin. Rheumatol* (2018) 37(1): 81-85. doi: 10.1007/s10067-017-3676-8

[pii];10.1007/s10067-017-3676-8.

40. Bernatsky S., Clarke A.E., Zahedi N.O., Labrecque J., Schanberg L.E., Silverman E.D., et al., Malignancy in Pediatric-onset Systemic Lupus Erythematosus. *J. Rheumatol* (2017) 44(10): 1484-1486. doi: jrheum.170179 [pii];10.3899/jrheum.170179.

41. Tallbacka K.R., Pettersson T., and Pukkala E., Increased incidence of cancer in systemic lupus erythematosus: a Finnish cohort study with more than 25 years of follow-up. *Scand. J. Rheumatol* (2018) 47(6): 461-464. doi: 10.1080/03009742.2017.1384054.

42. Kuo C.F., Chou I.J., Rees F., Grainge M.J., Lanyon P., Davenport G., et al., Temporal relationships between systemic lupus erythematosus and comorbidities. *Rheumatology. (Oxford)* (2019) 58(5): 840-848. doi: 5253846 [pii];10.1093/rheumatology/key335.

43. Bae E.H., Lim S.Y., Han K.D., Jung J.H., Choi H.S., Kim C.S., et al., Systemic lupus erythematosus is a risk factor for cancer: a nationwide population-based study in Korea. *Lupus* (2019) 28(3): 317-323. doi: 10.1177/0961203319826672 [doi].

44. Wang L.H., Wang W.M., Lin S.H., and Shieh C.C., Bidirectional relationship between systemic lupus erythematosus and non-Hodgkin's lymphoma: a nationwide population-based study. *Rheumatology. (Oxford)* (2019) 58(7): 1245-1249. doi: 5306646 [pii];10.1093/rheumatology/kez011.

45. Cobo-Ibez T., Urruticoechea-Arana A., Ra-Figueroa I., Martn-Martnez M.A., Ovalles-Bonilla J.G., Galindo M., et al., Hormonal Dependence and Cancer in Systemic Lupus Erythematosus. *Arthritis Care Res. (Hoboken.)* (2020) 72(2): 216-224. doi: 10.1002/acr.24068.

46. Nived O., Bengtsson A., Jensen A., Sturfelt G., and Olsson H., Malignancies during follow-up in an epidemiologically defined systemic lupus erythematosus inception cohort in southern Sweden. *Lupus* (2001) 10(7): 500-504. doi: 10.1191/096120301678416079.

47. Bernatsky S., Boivin J.F., Joseph L., Manzi S., Ginzler E., Gladman D.D., et al., Mortality in systemic lupus erythematosus. *Arthritis Rheum* (2006) 54(8): 2550-2557. doi: 10.1002/art.21955.

48. Lerang K., Gilboe I.M., Steinar T.D., and Gran J.T., Mortality and years of potential life loss in systemic lupus erythematosus: a population-based cohort study. *Lupus* (2014) 23(14): 1546-1552. doi: 0961203314551083 [pii];10.1177/0961203314551083.

49. Tselios K., Gladman D.D., Sheane B.J., Su J., and Urowitz M., All-cause, cause-specific and age-specific standardised mortality ratios of patients with systemic lupus erythematosus in Ontario, Canada over 43 years (1971-2013). *Ann. Rheum. Dis* (2019) 78(6): 802-806. doi: annrheumdis-2018-214802 [pii];10.1136/annrheumdis-2018-214802.

50. Bultink I.E.M., de V.F., van Vollenhoven R.F., and Lalmohamed A., Mortality, causes of death and influence of medication use in patients with systemic lupus erythematosus vs matched controls. *Rheumatology. (Oxford)* (2021) 60(1): 207-216. doi: 5870413 [pii];10.1093/rheumatology/keaa267.
